# Highly potent, naturally acquired human monoclonal antibodies against Pfs48/45 block *Plasmodium falciparum* transmission to mosquitoes

**DOI:** 10.1016/j.immuni.2023.01.009

**Published:** 2023-02-14

**Authors:** Amanda Fabra-García, Sophia Hailemariam, Roos M. de Jong, Kirsten Janssen, Karina Teelen, Marga van de Vegte-Bolmer, Geert-Jan van Gemert, Danton Ivanochko, Anthony Semesi, Brandon McLeod, Martijn W. Vos, Marloes H.C. de Bruijni, Judith M. Bolscher, Marta Szabat, Stefanie Vogt, Lucas Kraft, Sherie Duncan, Moses R. Kamya, Margaret E. Feeney, Prasanna Jagannathan, Bryan Greenhouse, Koen J. Dechering, Robert W. Sauerwein, C. Richter King, Randall S. MacGill, Teun Bousema, Jean-Philippe Julien, Matthijs M. Jore

**Affiliations:** 1 Department of Medical Microbiology, Radboudumc, Nijmegen, the Netherlands; 2 Program in Molecular Medicine, The Hospital for Sick Children Research Institute, Toronto, ON, Canada; 3 Department of Biochemistry, University of Toronto, Toronto, ON, Canada; 4 TropIQ Health Sciences, Nijmegen, the Netherlands; 5 AbCellera Biologics Inc., Vancouver, BC, Canada; 6 Infectious Disease Research Collaboration, Kampala, Uganda; 7 Department of Medicine, University of California, San Francisco, San Francisco, CA, USA; 8 Department of Pediatrics, University of California, San Francisco, San Francisco, CA, USA; 9 Department of Microbiology and Immunology, Stanford University, Stanford, CA, USA; 10 PATH's Malaria Vaccine Initiative, Washington, DC 20001, USA; 11 Department of Immunology, University of Toronto, Toronto, ON, Canada

**Keywords:** malaria, *Plasmodium falciparum*, human monoclonal antibodies, crystallography, Pfs48/45, transmission-reducing activity, transmission-blocking vaccine

## Abstract

Malaria transmission-blocking vaccines (TBVs) aim to induce antibodies that interrupt malaria parasite development in the mosquito, thereby blocking onward transmission, and provide a much-needed tool for malaria control and elimination. The parasite surface protein Pfs48/45 is a leading TBV candidate. Here, we isolated and characterized a panel of 81 human Pfs48/45-specific monoclonal antibodies (mAbs) from donors naturally exposed to *Plasmodium* parasites. Genetically diverse mAbs against each of the three domains (D1–D3) of Pfs48/45 were identified. The most potent mAbs targeted D1 and D3 and achieved >80% transmission-reducing activity in standard membrane-feeding assays, at 10 and 2 μg/mL, respectively. Co-crystal structures of D3 in complex with four different mAbs delineated two conserved protective epitopes. Altogether, these Pfs48/45-specific human mAbs provide important insight into protective and non-protective epitopes that can further our understanding of transmission and inform the design of refined malaria transmission-blocking vaccine candidates.

## Highlights

• Isolated 81 unique mAbs directed to Pfs48/45 elicited in naturally exposed individuals
• The most potent mAbs target domains 1 and 3 of Pfs48/45
• Antibodies against domain 2 have low or no transmission-reducing activity
• Potent antibodies against domain 3 target two conserved epitopes

## Graphical abstract

**Figure:**
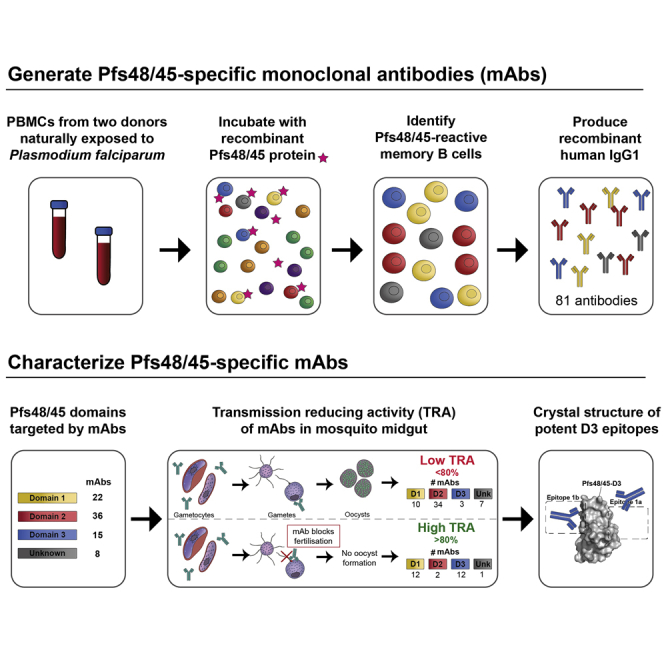

## Teaser

The malaria parasite surface protein Pfs48/45 is a leading transmission-blocking vaccine candidate, but little is known about the specificity of naturally acquired antibodies against this target. Fabra-García et al. isolate and characterize a panel of 81 human monoclonal antibodies from naturally exposed individuals and demonstrate that the most potent antibodies target domains 1 and 3 of Pfs48/45.

## Introduction

Malaria is a devastating disease caused by *Plasmodium* parasites that are transmitted by *Anopheles* mosquitoes. Despite intensive malaria control efforts, the number of malaria cases and related deaths have increased in recent years.^1^ Furthermore, the success of control programs is threatened by the emergence of artemisinin-resistant parasites in Africa^2^ and the spreading of insecticide-resistant mosquitoes.^1^ There is a broad consensus that novel tools, including tools that specifically block transmission, are needed to further reduce the burden of malaria and to continue progress toward malaria eradication.

*Plasmodium* transmission relies on the uptake of circulating gametocytes via a bloodmeal by female *Anopheles* mosquitoes. Inside the mosquito midgut, male and female gametocytes are activated and form gametes that egress from red blood cells to undergo fertilization. After fertilization, the parasites go through several developmental stages that lead to the formation of oocysts. Inside oocysts, sporozoites are formed that migrate to the salivary glands resulting in an infectious mosquito. Transmission-blocking vaccines (TBVs) aim to interrupt transmission from human to mosquito by inducing antibodies in humans that block parasite development inside the mosquito midgut and hence prevent onward transmission to other humans.^3^^,^^4^

**Figure 1:**
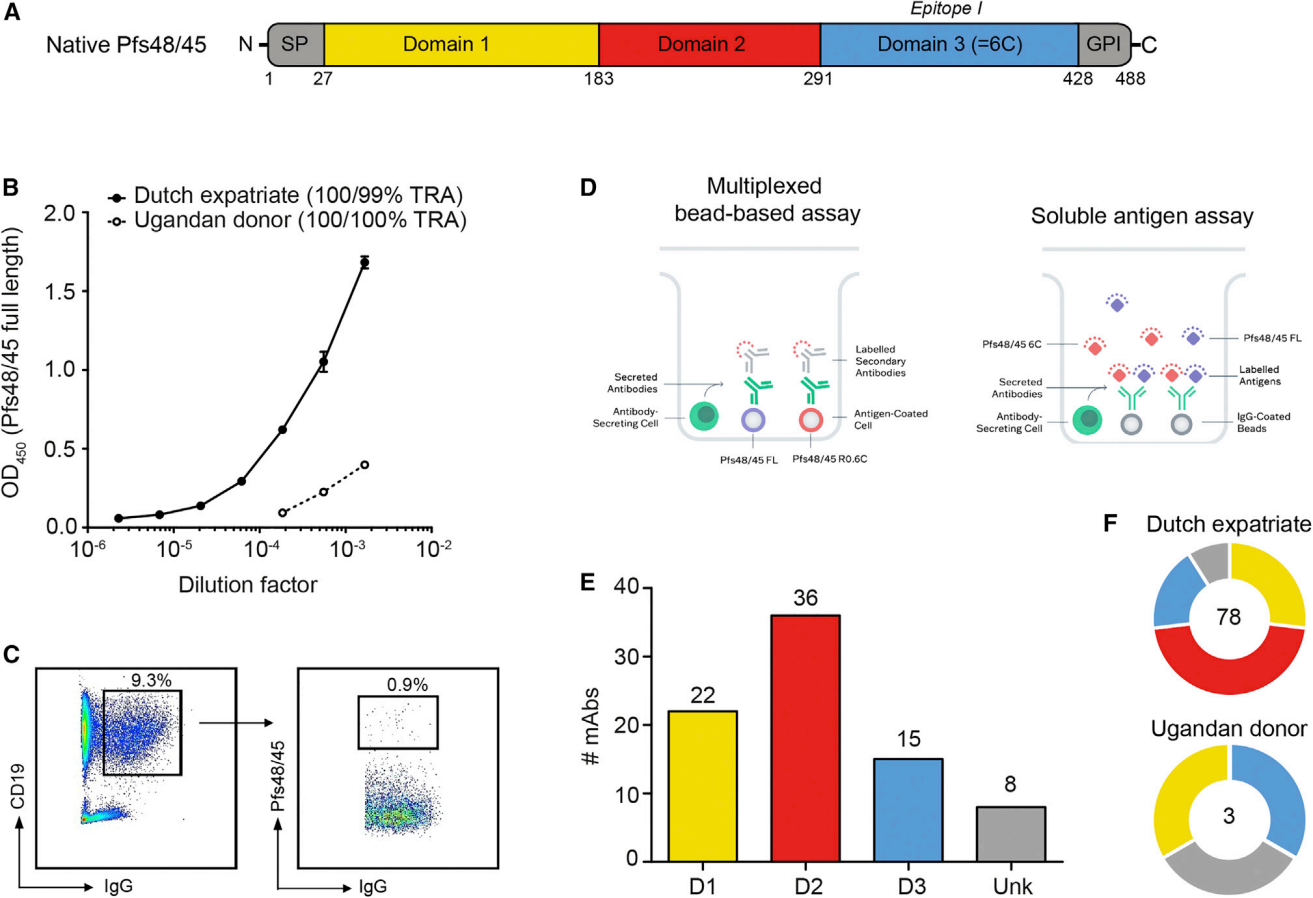
Isolation and domain specificity of 81 human Pfs45/45-specific mAbs (A) Schematic representation of Pfs48/45 that contains three 6-cysteine domains (domains 1–3), a signal peptide (SP), and a glycosylphosphatidylinositol anchor domain (GPI). Domain boundaries are indicated with amino acid numbers and follow predictions by Gerloff et al.^6^ Domain 3 is also known as “6C” and contains epitope I.^19^ (B) Recognition of full-length Pfs48/45 by plasma samples from two naturally exposed donors in an enzyme-linked immunosorbent assay. Values are means of two technical replicates and error bars represent the SEM. Transmission-reducing activity (TRA) of purified total IgG, tested at 1:3 dilution in the presence of complement, from both donors are shown in the legend and are outcomes of two independent standard membrane-feeding assays. The raw SMFA data are included in Table S7. (C) Gating strategy for antigen-specific sorting. Pfs48/45-specific B cells were isolated by gating CD19+, IgG+, and Pfs48/45+ cells from single live lymphocytes. (D) Schematic representation of multiplexed bead-based (left) and soluble antigen (right) screening assays on the microfluidic device. Memory B cells were activated *in vitro* into antibody-secreting cells and Pfs48/45 reactivity was determined using either antigen-coated beads and a fluorescently labeled secondary antibody (left) or anti-human IgG capture beads with fluorescently labeled Pfs48/45 antigens (right). (E) Summary of domain specificity of isolated mAbs (Table S1). Unk, unknown domain specificity. (F) Pie charts showing the number of isolated Pfs48/45-specific mAbs per donor (center) and fractions of D1-, D2-, and D3-specific mAbs in yellow, red, and blue. mAbs with unknown domain specificity are shown in gray. See also Figures S1–S3 and Table S1.

Pfs48/45 is a leading TBV candidate, present on the surface of late-stage gametocytes and activated gametes, and comprises three 6-cysteine domains and a glycosylphosphatidylinositol (GPI) anchor^5^^,^^6^ (Figure 1A). Parasites that lack Pfs48/45 fail to generate oocysts, and rodent malaria parasites without P48/45 produce infertile male gametes, strongly suggesting that Pfs48/45 plays a key role in gamete fertilization.^7^ Pfs48/45-specific rodent monoclonal antibodies (mAbs), raised against whole parasites, can prevent oocyst formation^8^^,^^9^^,^^10^—this formed the basis for the development of Pfs48/45 as a TBV candidate. The most potent transmission-blocking mAb described to date is 85RF45.1,^10^ which targets the conserved epitope I on Pfs48/45 domain 3 (D3, also known as Pfs48/45-6C) and blocks the transmission of genetically diverse *Plasmodium falciparum* (*P. falciparum*) strains.^11^^,^^12^ A humanized version, TB31F, has recently been generated^11^ and has completed early clinical evaluation.^13^ The pre-clinical development of a Pfs48/45-based TBV has long been hampered by difficulties in producing correctly folded antigen (reviewed in Theisen et al.^14^). However, there has been considerable progress recently with the expression of D3-containing constructs in *Lactococcus lactis* (R0.6C and ProC6C, where 6C denotes D3)^15^^,^^16^^,^^17^ and full-length Pfs48/45 in *Drosophila melanogaster* S2 cells.^18^ These vaccine candidates are currently being evaluated in phase I clinical trials (Clinicaltrials.gov IDs: NCT04862416, NCT05400746).

Pfs48/45 is expressed on the surface of gametocytes that, although located inside red blood cells, are circulating in the human bloodstream. The clearance of gametocyte-infected red blood cells by the spleen can expose this antigen to the human immune system, resulting in the natural acquisition of antibodies in gametocyte-carrying individuals (reviewed in Stone et al.^20^). We recently purified Pfs48/45-specific polyclonal antibodies from naturally exposed donors and demonstrated their ability to block transmission of cultured gametocytes in the standard membrane-feeding assay (SMFA).^21^ Together, these data demonstrate that Pfs48/45 is immunogenic and can induce naturally acquired functional antibodies in *Plasmodium*-infected humans, but little is known about the mAbs that make up the response. These mAbs could provide valuable insights into functional and non-functional epitopes and thereby inform vaccine design. Furthermore, potent human mAbs may also be considered for passive immunization strategies.^13^^,^^22^^,^^23^^,^^24^

Here, we isolated human Pfs48/45-specific mAbs from memory B cells (MBCs) of two naturally exposed donors with high serum transmission-reducing activity (TRA). We determined the domain and epitope specificity of these mAbs and linked these to functional activity. Finally, we used X-ray crystallography to delineate epitopes of potent D3-specific mAbs, providing atomic insights into mAb functional activity.

## Results

### Isolation of Pfs48/45-specific MBCs from naturally exposed donors

For the isolation of Pfs48/45-specific mAbs, we selected two donors that had experienced repeated *Plasmodium* infections. One donor was a 69-year-old Dutch expatriate who had lived in central Africa for approximately 30 years. This donor had total IgG that recognized full-length Pfs48/45 in ELISA and strongly reduced transmission (Figure 1B; donor A in Stone et al.^21^). We selected the other donor from a panel of 1,358 donors from Tororo, an area with high malaria transmission in Uganda with an estimated exposure of 310 *P. falciparum*-infected mosquito bites per person per year.^25^ Plasma samples from these donors were screened for (1) high antibody titers against gametocyte extract, (2) high TRA of purified IgGs in SMFA, and (3) the presence of antibodies against Pfs48/45. The total IgG of the selected 8-year-old Ugandan donor showed 100% TRA in SMFA and recognized full-length Pfs48/45 by ELISA, albeit to a lesser extent than that of the Dutch expatriate (Figure 1B). Using fluorescently labeled full-length Pfs48/45, we sorted 123 single MBCs from the Dutch expatriate and obtained 46 unique paired antibody sequences (Figure 1C). We also used a microfluidic device to screen single MBCs from both donors for Pfs48/45 reactivity (Figure 1D). This screening method identified 601 Pfs48/45-specific hits from which 91 unique paired antibody sequences were obtained. We recombinantly expressed 100 unique antibodies, obtained from one or both of the B cell screening methods, as human IgG1 to confirm specificity to Pfs48/45. Eighty-one mAbs bound to full-length recombinant Pfs48/45 in ELISA (Table S1) or showed high-affinity binding by surface plasmon resonance (SPR) (Figure S1A), and 74 of these recognized Pfs48/45 in gametocyte extract by western blot (Figure S2A). All 81 mAbs recognized Pfs48/45 in its native configuration on the female gamete surface membrane as detected by surface immunofluorescence assay (Figure S2B). Altogether, we isolated 81 Pfs48/45-specific mAbs, which recognized the surface of female gametes, from two naturally exposed donors.

### Isolated mAbs target different domains of Pfs48/45

To determine the domain specificity of Pfs48/45-specific mAbs, we produced three Pfs48/45 protein fragments that contained D1-2, D2-3, and D3 only, respectively (Figures S1B and S1C). Previously described rodent mAbs^10^ recognized these fragments in western blot and ELISA, confirming that the fragments contained domains that were properly folded (Figures S1D and S1E). Using these protein fragments, we tested the binding of the 81 human mAbs in ELISA and found that 22 antibodies bound to D1, 36 bound to D2, and 15 bound to D3 (Figure 1E; Table S1). Seven mAbs did not show reactivity with any of the fragments. One mAb, RUPA-154, reacted with all three constructs, suggesting it targets an epitope that spans multiple domains. Eleven out of 22 D1-specific mAbs bound to native Pfs48/45 protein under reducing conditions and thus target an epitope that is primarily linear (Figure S2A). The other mAbs, including all D2- and D3-specific antibodies, lacked reactivity under reducing conditions and therefore target more conformational epitopes (Figure S2A). Having established domain specificity, we next mapped the fine specificity of the human mAbs in competition experiments, which included previously described rodent mAbs^10^^,^^26^ and the highly potent humanized mAb TB31F^11^ as reference mAbs. mAbs fell into 42 bins that could be grouped into four larger clusters (Figure S3A). The first cluster contains previously identified D1-specific rat mAb 85RF45.5, the second contains D2-specific rat mAb 85RF45.3, the third contains D3-specific rat mAb 85RF45.1, mouse mAb 32F3, and the humanized mAb TB31F, whereas the fourth lacks reference mAbs. Competition patterns within clusters are complex and clusters show extensive interactions with each other, suggesting a high diversity in epitope specificity (Figure S3A). The clusters defined by competition analyses align well with the domain specificity determined by ELISA (Figure S3B). Some D1-specific mAbs compete with D3-specific mAbs, suggesting that D1 and D3 may be in close relative proximity to each other in the full-length protein, or that the epitopes may be allosterically interconnected. Altogether, we identified 81 human mAbs that cover a range of specificities: 78 were obtained from the Dutch expatriate and 3 were obtained from the Ugandan donor (Figure 1F), consistent with the difference in observed antibody titers in plasma.

### Potent transmission-blocking mAbs bind Pfs48/45-D1 and D3

**Figure 2:**
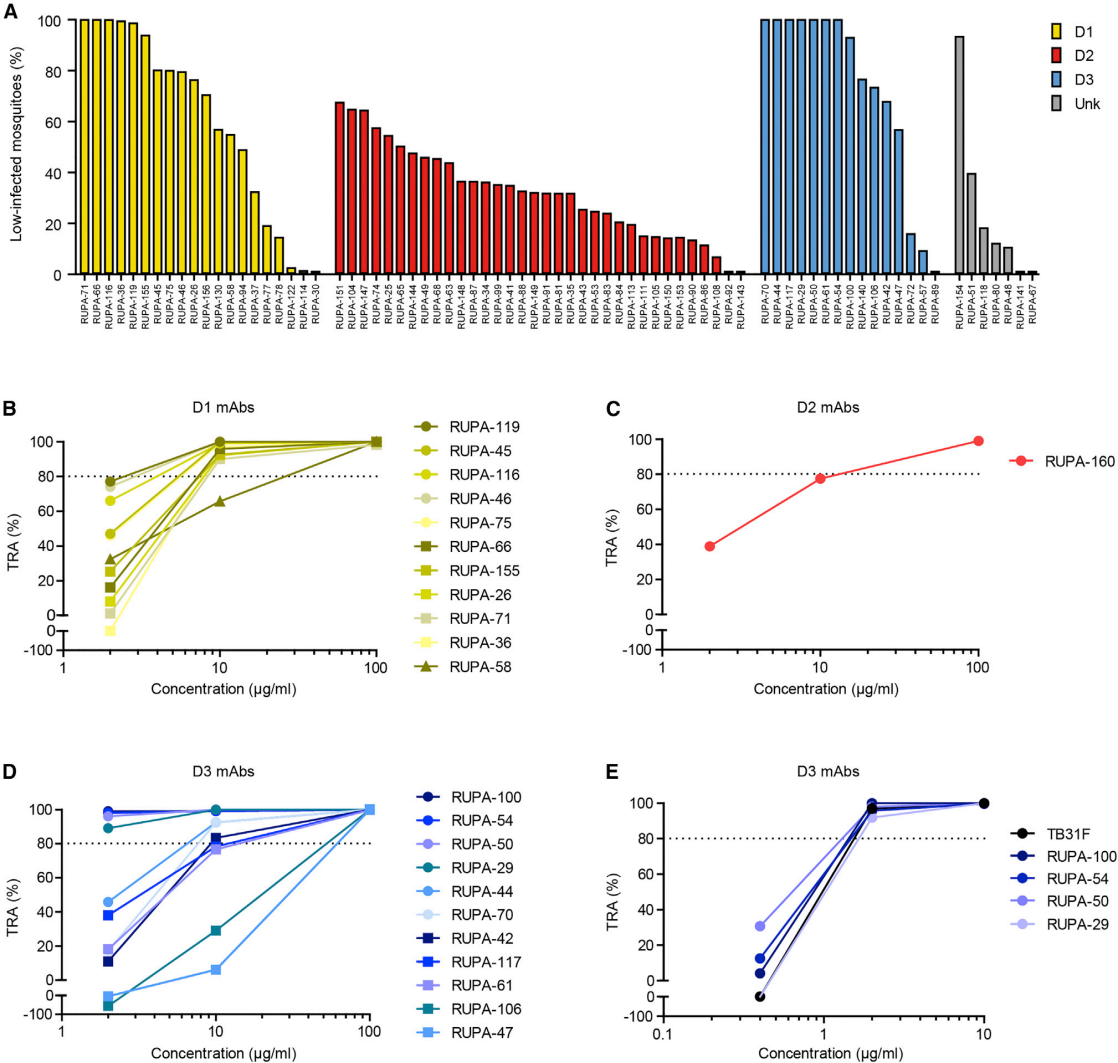
Potent mAbs target Pfs48/45 D1 and D3 (A) mAbs were tested at 100 μg/mL in a barcoded membrane-feeding assay using *Anopheles stephensi* mosquitoes and transgenic *Plasmodium falciparum* NF54 parasites that express a luciferase reporter.^27^ The figure shows the proportion of mosquitoes with low infection (>90% reduction in oocyst intensity relative to the vehicle controls) 8 days after feeding. Note that five mAbs, including RUPA-160, were not available at the time the barcoded membrane-feeding assay was performed and were only tested in a standard membrane-feeding assay (Figure S3D). (B–D) D1-specific (B), D2-specific (C), and D3-specific (D) mAbs that showed >95% TRA in standard membrane-feeding assay (SMFA) at 100 μg/mL (Figure S3D) were titrated to determine their potency. (E) The most potent D3-specific mAbs were further titrated and tested head-to-head with TB31F in the single SMFA experiment. mAbs are colored according to domain specificity and TRA values (B–E) were based on single SMFA experiments with 20 mosquitoes per condition and calculated as the percentage reduction in oocyst intensity compared with a negative control. Raw SMFA data and 95% confidence intervals are presented in Table S7. See also Figure S3.

To determine the functional potency of the human mAbs, we tested these in a series of membrane-feeding assays with cultured *P. falciparum* NF54 gametocytes and *Anopheles stephensi* mosquitoes. We first screened the mAbs at 100 μg/mL in a barcoded membrane-feeding assay that quantifies the percentage of low-infected mosquitoes to identify mAbs with strong TRA.^27^ In this assay, the mAbs displayed a wide range of activities—the most potent mAbs target D1 and D3, whereas most of the D2-specific mAbs showed weak TRA (Figure 2A). To confirm high potency, we next tested the 36 most potent mAbs in SMFA. We also included five mAbs that were not yet available at the time of the high-throughput membrane-feeding assay. Twenty-seven mAbs showed more than 80% reduction in oocyst intensity at 100 μg/mL, including 12 D1-, two D2-, and 12 D3-specific mAbs and one mAb with unknown domain specificity (Figure S3D). This represents 55% (12/22), 6% (2/36), and 80% (12/15) of all unique D1-, D2-, and D3-specific mAbs, respectively. We then titrated mAbs that showed ˃95% TRA at 100 μg/mL to determine their potency in more detail (Figures 2B–2D). D1-specific mAbs, except RUPA-58, were similarly potent with IC_80_ values between 2 and 10 μg/mL, D2-specific RUPA-160 showed approximately 80% TRA at 10 μg/mL, whereas D3-specific mAbs showed a larger range of potencies. Four D3-specific mAbs, RUPA-29, -50, -54, and -100, showed more than 80% TRA at a low concentration of 2 μg/mL, similar to that of the most potent transmission-blocking antibody described to date, TB31F (Figure 2E). Together, these data demonstrate that natural *Plasmodium* infection can induce functional antibodies against all three domains of Pfs48/45, that strong functional activity is observed for mAbs targeting both D1 and D3, and that the most potent mAbs target D3.

### Active mAbs are genetically diverse

**Figure 3:**
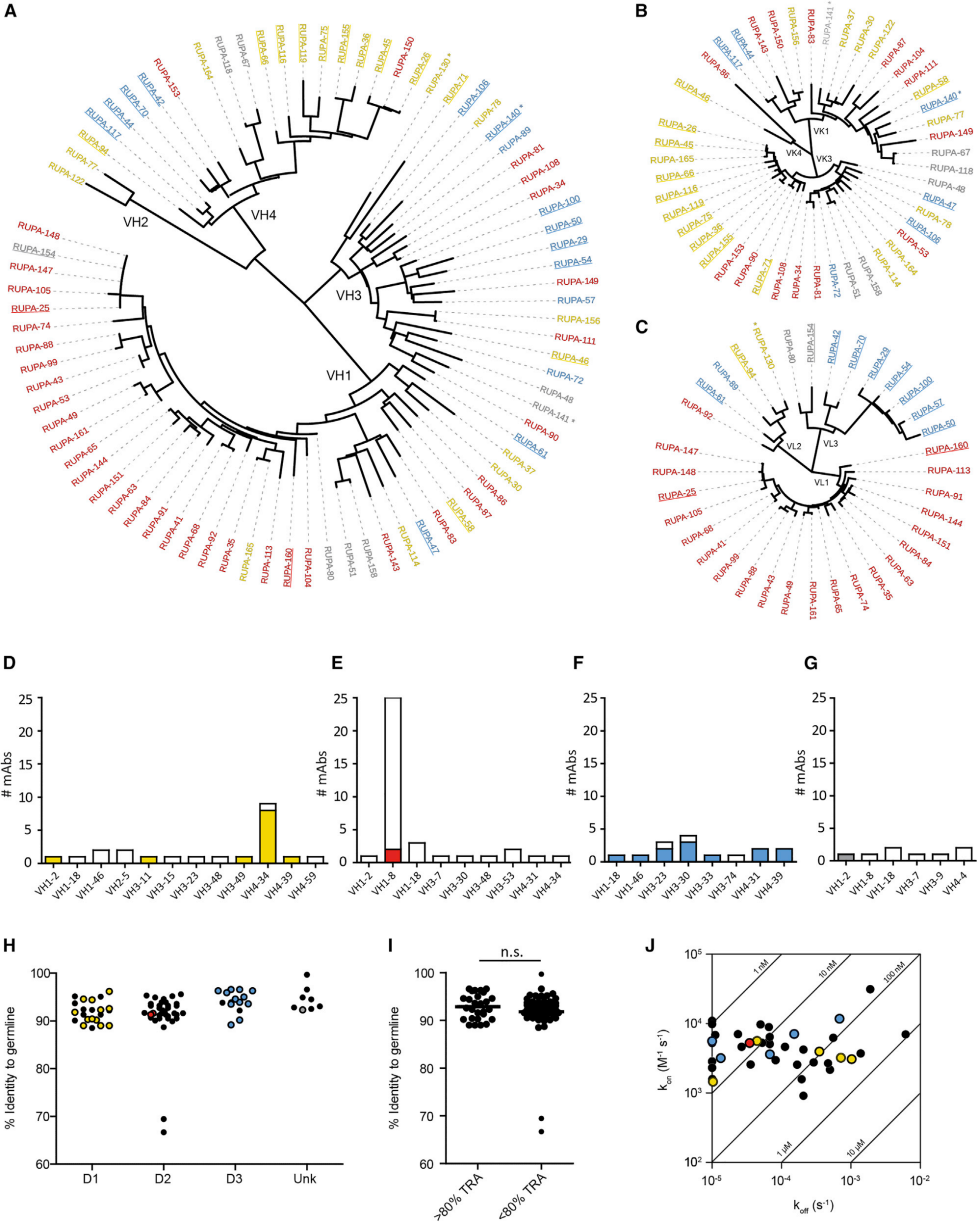
Pfs48/45-specific mAbs are genetically diverse (A–C) Phylogenetic trees of (A) VH, (B) VK, and (C) VL chain sequences. mAbs are colored according to domain specificity. Potent mAbs, i.e., >80% TRA in SMFA, are underlined. mAbs from the Ugandan donor are marked with an asterisk. (D–G) Bar graphs showing the number of mAbs per heavy-chain family for (D) D1-, (E) D2-, and (F) D3-specific mAbs and (G) mAbs with unknown specificity. Colored bars represent mAbs with >95% TRA at 100 μg/mL with the actual color-reflecting domain (as in Figure 1). (H) Graph showing heavy-chain gene sequence identity to germline sequences. Individual dots represent individual mAbs and are grouped by domain specificity. Colored dots are mAbs with >95% TRA at 100 μg/mL. (I) mAbs grouped by potency. Groups are compared by Mann-Whitney test. n.s., not significant. (J) Isoaffinity plot showing binding kinetics as determined by surface plasmon resonance with immobilized antibodies and full-length Pfs48/45 as analyte. Colored dots are mAbs with >80% TRA at 100 μg/mL and are colored by domain specificity. k_on_, association constant; k_off_, dissociation constant. See also Figure S3 and Table S2.

The heavy and light chains of the Pfs48/45-specific mAbs display a large genetic diversity (Figures 3A–3C; Table S2). Heavy-chain sequences originate from 50 different B cell lineages and use 21 different VH segments; D1-specific mAbs are encoded by 12 different VH segments, D2-specific mAbs by eight, and D3-specific mAbs by nine different VH genes (Figures 3D–3G); and 46 out of 81 (57%) of the mAbs contained a kappa light chain (Figures S3E–S3L; Table S2). The mAbs isolated from the Ugandan donor were genetically distinct from those acquired from the Dutch expatriate donor (Table S2). The two most expanded VH families were IGHV1-8 and IGHV4-34, comprising 26 mAbs that target D2 and nine mAbs that target D1, respectively (Figures 3D and 3E). One of these families, IGHV1–8, contained the only two D2-specific mAbs (RUPA-25 and RUPA-160) with high potency in SMFA (i.e., >80% TRA at 100 μg/mL; Figure S3D). Although these mAbs share the same heavy- and light-chain gene segments with many other mAbs from this family, the complementarity-determining region 3 (CDR3) sequences are different, suggesting that the CDR sequences determine functional TRA. Although the majority of high potency mAbs that target D1 were genetically similar and contained VH4-34 segments, potent mAbs that target D3 were more genetically diverse. Although the heavy chains of the four most potent D3 antibodies (RUPA-29, RUPA-50, RUPA-54, and RUPA-100) are encoded by the related IGHV3-30 and IGHV3-33 germline genes, the light chains of these antibodies are encoded by the same lambda chain variable domain. Somatic hypermutations were generally low and similar across domain specificities (Figure 3H). Potent mAbs did not contain more somatic hypermutations than other mAbs (p = 0.58) (Figure 3I). The binding affinity of the mAbs ranges from the low nanomolar to micromolar range (Figures 3J and S1A). Although we did not obtain affinity data for all mAbs, high potency mAbs did not have higher affinities nor lower off- or higher on-rates (Figures 3J and S1). Together, these data demonstrate that the isolated mAbs are genetically diverse and that potency is not only determined by genetic origin, affinity maturation, or binding affinity.

### Structural characterization of potent epitope Ia mAbs

To date, our molecular understanding of *P. falciparum* transmission inhibition as mediated by antibody binding to Pfs48/45-D3 has been limited to crystal structures of D3 in complex with three Fabs: 85RF45.1 (PDB: 6H5N and 6E62), TB31F (PDB: 6E63), and 32F3 (PDB: 7ZWI), which all target epitope I on D3.^11^^,^^18^^,^^28^ To expand on this knowledge, we solved structures of two ternary complexes: D3 bound to RUPA-47 Fab and RUPA-117 Fab, and D3 bound to RUPA-29 Fab and RUPA-44 Fab, at resolutions of 2.18 and 2.86 Å, respectively (Figures S4A-S4D; Table S3; PDB: 7UNB).^29^

**Figure 4:**
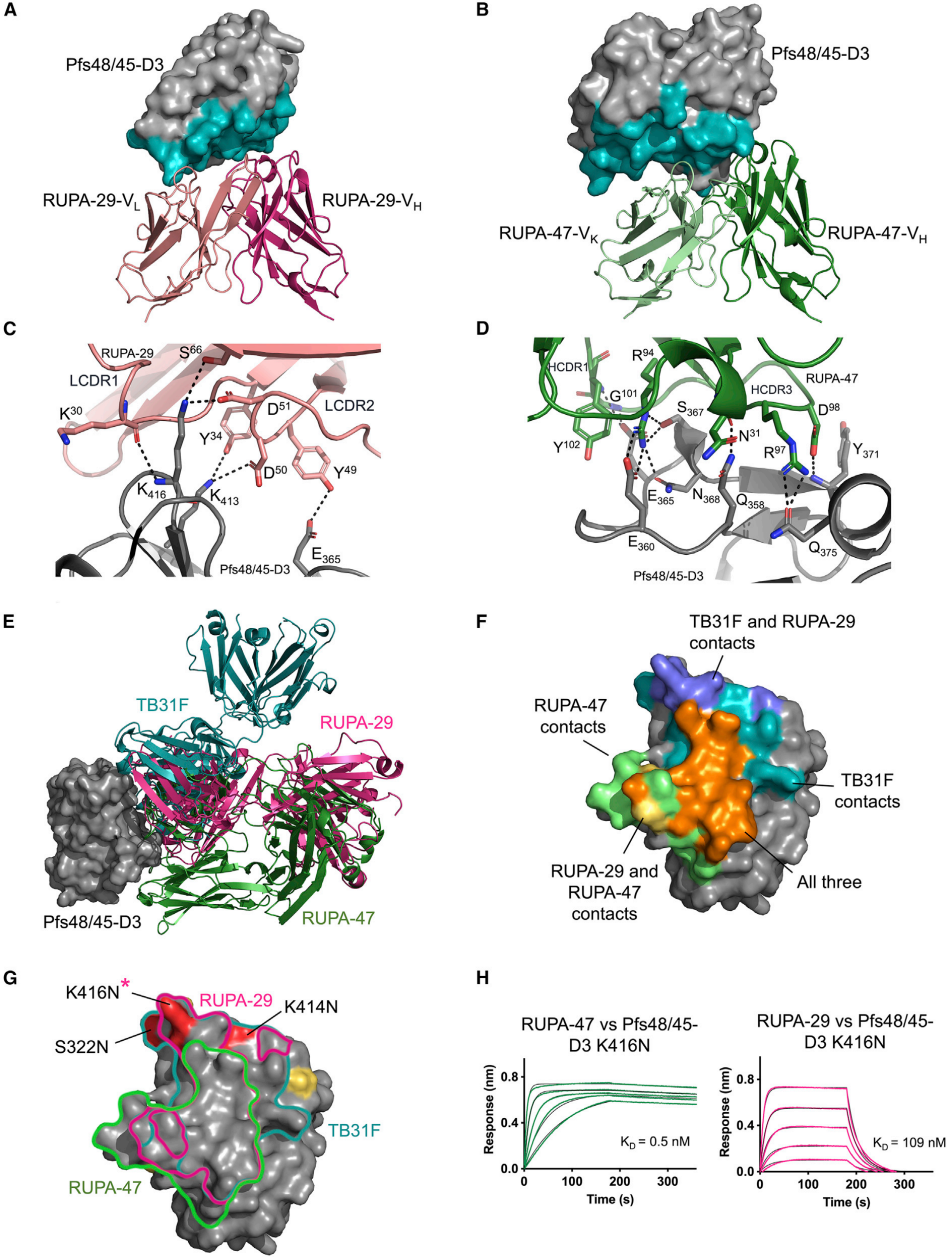
Delineation of Pfs48/45-D3 epitope Ia recognized by human antibodies (A and B) Variable domains of (A) RUPA-29 and (B) RUPA-47 bound to Pfs48/45-D3. Pfs48/45-D3 is depicted as the surface with the epitope of TB31F colored in teal. (C and D) Interactions between Pfs48/45-D3 and RUPA-29 or RUPA-47. RUPA-29, RUPA-47, and Pfs48/45-D3 are depicted as cartoons with residues forming H-bonds and salt bridges (black dashes) shown as sticks. (C) Interactions between LCDRs of RUPA-29 and Pfs48/45-D3. (D) Interactions between HCDRs of RUPA-47 and Pfs48/45-D3. (E) Structure of Pfs48/45-D3 (gray) bound to RUPA-29 (pink), RUPA-47 (green), and TB31F (teal; PDB: 6E63). (F) Pfs48/45-D3 in gray with residues that contact only TB31F (teal), only RUPA-47 (green), both RUPA-29 and RUPA-47 (yellow), both TB31F and RUPA-29 (slate), and all three (orange). (G) Pfs48/45-D3 shown as the surface with the epitopes of RUPA-47 (green), RUPA-29 (pink), and TB31F (teal) outlined. Polymorphisms that do not interact with mAbs are in yellow, whereas those that interact with mAbs are in red and labeled. Polymorphisms that impact antibody binding are indicated with an asterisk. (H) Biolayer interferometry (BLI) curves of Fabs RUPA-47 and RUPA-29 binding to Pfs48/45-D3 with the K416N polymorphism. See also Figures S4 and S5 and Tables S3–S5.

First, to structurally characterize this epitope I in more detail, we examined the co-crystal structures of two potent antibodies that compete with TB31F, RUPA-29 (TRA of >80% at 2 μg/mL), and RUPA-47 (TRA of 100% at 100 μg/mL that drops to 20% at 10 μg/mL), as Fabs bound to D3 (Figures 4A, 4B, S3C, and S4A–S4D; Table S3; PDB: 7UNB). RUPA-29 is one of several genetically similar antibodies with >80% TRA at 2 μg/mL, whereas RUPA-47 is highly inhibitory but less potent than some of the other D3 binders, making both antibodies informative for delineating epitope 1 (Figures 2D and 3A).

RUPA-29 binds primarily to β strands d and d′ (residues 347–356), β strand d″ (residues 368–371), and the intervening loop between β strands g and h (residues 413–416) of D3. Its interactions with D3 are mediated by all three CDRs of both the heavy chain (buried surface area [BSA] = 382 Å^2^) and the light chain (BSA = 312 Å^2^, total BSA = 694 Å^2^) (Table S4). The light-chain forms four H-bonds with D3 mediated by antibody residues K^30^, Y^34^, Y^49^, and S^66^, and two salt bridges mediated by residues D^50^ and D^51^ (Figure 4C). The heavy chain provides an additional four H-bonds from heavy-chain CDR1 (HCDR1) and HCDR2, and van der Waals interactions formed by HCDR3 residues including F^96^ (54 Å^2^), H^98^ (68 Å^2^), and F^100A^ (47 Å^2^) (Table S4). RUPA-29, along with RUPA-100, RUPA-54, and RUPA-50, is part of a highly potent antibody lineage made up of an IGHV3-33 or IGHV3-30 heavy chain and an IGLV3-10 lambda chain. An alignment of the lambda-chain CDRs (LCDRs) of these four mAbs revealed that most of the RUPA-29 contact residues in the RUPA-29-D3 structure, including those that form electrostatic interactions with D3, are well conserved (Figures S5A–S5D; Table S4). Although the HCDR residues involved in RUPA-29 binding to D3 are more variable across this antibody lineage, these differences mainly occur at residues involved in van der Waals interactions that can be mediated by several different amino acids (Figures S5A–S5D; Table S4).

RUPA-47 forms extensive interactions with D3, with a total BSA of 1,005 Å^2^ (Table S4). Both the heavy chain and light chain contribute to binding, with BSAs of 557 and 448 Å^2^, respectively. RUPA-47’s heavy chain interacts extensively with loop 357–369 of Pfs48/45-D3 through H-bonds and salt bridges formed by HCDR1 and HCDR3 residues N^31^, R^94^, G^101^, and Y^102^ (Figure 4D). Kappa-chain CDR1 (KCDR1) and KCDR2 residues R^29^, Y^32^, and S^52^ of the RUPA-47 light-chain form a salt bridge and several H-bonds with D_351_, Q_355_, and K_413_ of Pfs48/45-D3 (Table S4).

An overlay of the RUPA-47-bound and RUPA-29-bound D3 structures with the TB31F-bound D3 structure reveals that their epitopes overlap considerably with one another (Figures 4E and 4F). This finding is consistent with both RUPA-47 and RUPA-29 competing with TB31F in binding competition assays (Figure S3C). RUPA-29 and TB31F share the bulk of their key contacts on D3 and their variable domains are positioned similarly with regard to D3. Pfs48/45 residues that form salt bridges and H-bond with RUPA-29 and TB31F are largely shared (D_351_, Q_355_, Y_371_, K_413_, and K_416_) (Figure 4F). RUPA-47 and TB31F bind overlapping but slightly different sites, with RUPA-47 interacting more heavily with loop 357–369 of D3 and having a slightly different angle of approach (Figures 4E and 4F). All three antibodies bind D3 with nanomolar binding affinities, with K_D_’s of 3.7, 0.4, and 0.3 nM for TB31F,^11^ RUPA-29, and RUPA-47, respectively (Table S5). Given that these are all strong binders to the recombinant protein, the lower inhibitory potency of RUPA-47 compared with TB31F and RUPA-29 may result from its different angle of approach or its epitope footprint being shifted toward loop 357–369 of D3. Overall, we structurally delineate this potent epitope bin as Ia.

Twelve non-synonymous single-nucleotide polymorphisms (SNPs) with varying frequencies have been identified in D3 across *P. falciparum* isolates.^30^ These include V304I/D, L314I, D320H, S322N, P359A, I376L, A387T, K414N, K416N, T422K, and T436I. Out of these SNPs, three occur in epitope Ia (S322N, K414N, and K416N) (Figure 4G). The S322N mutation is relatively common, with a frequency of 39.0%, whereas both K414N and K416N mutations are very rare, with frequencies of 0.007% and 0.03%, respectively.^30^ RUPA-47 does not interact with any of these residues, whereas RUPA-29 forms a salt bridge and two H-bonds with K_416_ through light-chain residues D^51^, S^66^, and K^30^. Kinetics experiments of Fabs RUPA-47 and RUPA-29 binding to a D3 construct containing the K416N mutation showed that RUPA-47’s binding affinity remains unchanged, whereas RUPA-29’s binding affinity drops with the introduction of this rare polymorphism but remains in the nanomolar affinity range (109 nM; Figure 4H; Table S5). Together, these results indicate that the epitope Ia antigenic site on D3 is largely conserved and can be recognized by potent human antibodies resilient to SNPs reported in field isolates.

### Structural delineation of potent epitope Ib on Pfs48/45-D3

**Figure 5:**
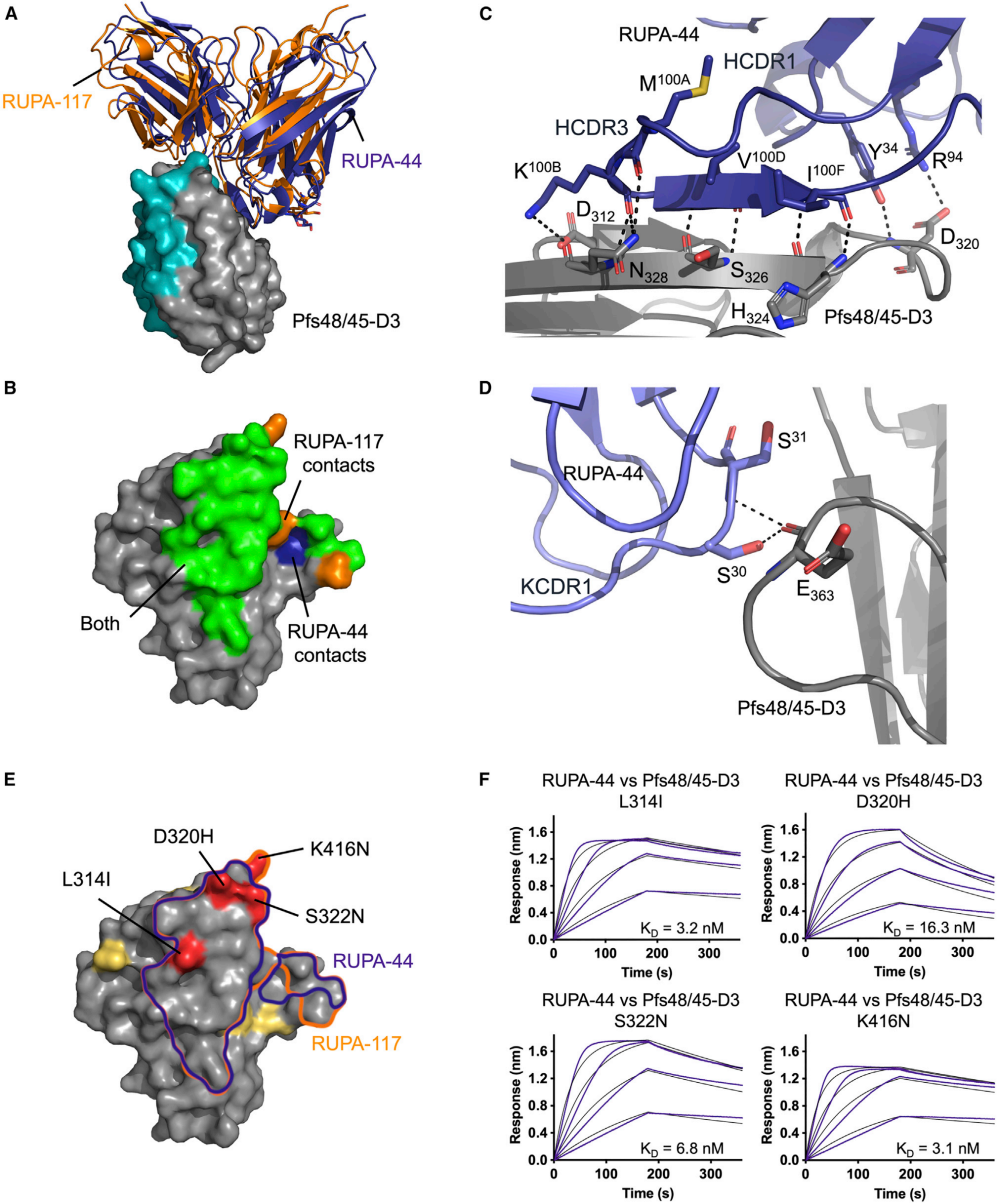
Structural delineation of potent epitope Ib on Pfs48/45-D3 (A) Surface representation of Pfs48/45-D3 (gray) bound to RUPA-44 (dark blue) and RUPA-117 (orange). The TB31F epitope on Pfs48/45-D3 is colored in teal. (B) Pfs48/45-D3 surface colored according to contact residues from only RUPA-44 (dark blue), only RUPA-117 (orange), both (green), and neither (gray). (C and D) Interactions between RUPA-44 and Pfs48/45-D3. RUPA-44 and Pfs48/45-D3 are depicted as cartoons with residues forming H-bonds and salt bridges (black dashes) shown as sticks. (C) Detailed interactions between RUPA-44 HCDR3 and HCDR1 residues and Pfs48/45-D3. (D) Detailed interactions between RUPA-44 KCDR1 residues and Pfs48/45-D3. (E) Pfs48/45-D3 shown as the surface with the epitopes of RUPA-44 (purple) and RUPA-117 (orange) outlined. Polymorphisms are indicated in yellow if they do not interact with mAbs and in red and labeled if they do. (F) Biolayer interferometry (BLI) curves of Fab RUPA-44 binding to Pfs48/45-D3 constructs with single mutations L314I, D320H, S322N, and K416N. See also Figures S4 and S5 and Tables S3, S5 and S6.

Using X-ray crystallography, we next uncovered the epitopes of non-TB31F-competing, potent antibodies RUPA-44 and RUPA-117 (Figures 5A, 5B, and S4A–S4D; Table S3, PDB: 7UNB).^29^ RUPA-44 and RUPA-117 have almost identical sequences, with just two amino acid substitutions in the light chain and four in the heavy chain (Figure S5E). As a result, they bind to very similar epitopes and share the majority of contacts. RUPA-44 has a BSA of 841 Å^2^, with the heavy chain and the light chain contributing 544 and 297 Å^2^, respectively, whereas RUPA-117 has a BSA of 795 Å^2^, with the heavy chain and the light chain contributing 522 and 273 Å^2^, respectively (Table S6). Most of the interactions between these two antibodies and D3 are mediated by their HCDR3 loop (RUPA-117 = 402 Å^2^, RUPA-44 = 414 Å^2^ BSA). The 19-residue HCDR3 of the antibodies forms a β-hairpin that interacts with β strand b of the D3 β sandwich (residues 324–331) (Figure 5C). Both RUPA-44 and RUPA-117 have the same HCDR3 sequence. HCDR3 residues R^94^, M^100A^, K^100B^, V^100D^, and I^100F^ form H-bonds and salt bridges with D3 residues D_320_, D_312_, H_324_, S_326_, and N_328_ (Figure 5C). The light-chain KCDR1 contributes additional H-bonds mediated by S^30^ and S^31^ for RUPA-44, and S^28^, S^30^, and I^31^ for RUPA-117 (Figure 5D). Despite these antibodies competing minimally with epitope Ia binders (Figure S3C), there is a slight overlap between epitope Ia and epitope Ib. Indeed, five residues in RUPA-117’s epitope (D_321_, S_322_, E_362_, E_363_, and K_416_) and five residues in RUPA-44’s epitope (D_321_, S_322_, E_362_, E_363_, and L_364_) are also part of epitope Ia.

The epitope Ib is relatively conserved in *P. falciparum* isolates, with only four reported SNPs (L314I, D320H, S322N, and K416N) of 28.2%, 0.007%, 39.0%, 0.03% frequencies, respectively (Figure 5E).^30^ Isolates containing both the S322N and L314I SNPs have also been found with a frequency of 1.1%.^30^ RUPA-44 and RUPA-117 make van der Waals interactions with L_314_ and S_322_. Both also form a salt bridge with D_320_ and a H-bond with its backbone amide. Additionally, RUPA-117 also forms van der Waals interactions with K_416_. Binding kinetics experiments with D3 constructs containing the single L314I, D320H, S322N, and K416N point mutations revealed that none of these mutations substantially impact RUPA-44 or RUPA-117 binding—both Fabs bound all antigens with affinities <20 nM (Figure 5F; Table S5). We therefore structurally delineate an antigenic site on D3, which can be recognized by potent human antibodies that can accommodate known SNPs.

## Discussion

Here, we isolated highly potent human Pfs48/45 mAbs from individuals naturally exposed to *Plasmodium*. We characterized the binding and epitope specificity of 81 mAbs and linked these to functional activity, providing insight into functional and non-functional human antibody responses toward this leading TBV candidate.

Naturally acquired TRA occurs after exposure to circulating *Plasmodium* gametocytes that are not taken up by mosquitoes but cleared by the spleen. Strong functional TRA is a rare phenotype that is not predicted by cumulative exposure or increasing age and remains poorly characterized.^21^ Several studies have demonstrated the presence of naturally acquired antibodies to Pfs48/45 in humans and its association with TRA (reviewed in Stone et al.^20^). We recently demonstrated causality for this association—affinity-purified naturally acquired antibodies to D2 and D3 blocked transmission in SMFA.^21^ Here, we provide insight into mAbs that make up polyclonal responses in humans and identified mAbs against all three domains of Pfs48/45. Many of these mAbs showed weak or negligible activity, particularly those that bind to D2. However, we identified 26 potent mAbs with more than 80% TRA at 100 μg/mL that mostly targeted D1 and D3. A similar pattern has been observed for rodent mAbs against Pfs48/45.^10^^,^^18^^,^^31^ Potent mAbs did not have more somatic hypermutations or higher affinity, further supporting the hypothesis that the target epitope is the main determinant of functional activity.

**Figure 6:**
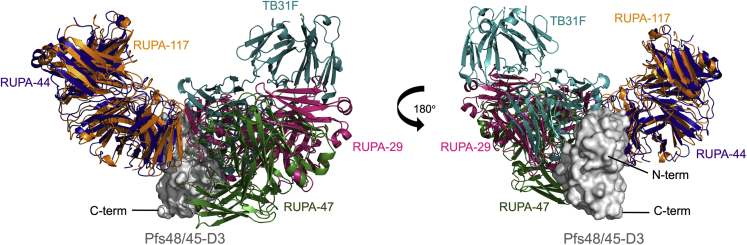
Potent inhibitory antibodies recognize one face on Pfs48/45-D3 Pfs48/45-D3 shown as surface (gray) bound to Fabs RUPA-44 (dark blue), RUPA-117 (orange), TB31F (teal; PDB: 6E63), RUPA-29 (pink), and RUPA-47 (dark green).

Since the most potent mAbs bound to D3, many with 80%–100% TRA at or below 2 μg/mL, we characterized their interactions at a molecular level. Our epitope binning experiments revealed two protective epitopes on D3—potent antibodies competed with either TB31F, RUPA-117, or both. Structures of Pfs48/45-D3-Fab complexes were solved using X-ray crystallography to delineate the epitopes of four antibodies (RUPA-44, RUPA-117, RUPA-47, and RUPA-29). The structures of TB31F-competing human antibodies RUPA-29 and RUPA-47 allowed for a more detailed look at the Pfs48/45 epitope historically referred to as epitope I. A relatively wide range of TRA observed for the TB31F-competing antibodies suggests that the now-expanded epitope bin Ia antigenic site may be associated with subtle differences in antibody recognition that considerably impact potency, the molecular basis of which still needs to be fully uncovered. Out of the antibodies isolated, many share genetic similarities. RUPA-29 is one of several potent mAbs that have an IGHV3-33 or IGHV3-30 heavy chain paired with an IGLV3-10 lambda chain (RUPA-29, RUPA-100, RUPA-54, and RUPA-50). An alignment of the heavy chains and lambda chains of RUPA-100, RUPA-50, and RUPA-54 with RUPA-29 revealed that most of the lambda chain contact residues in the RUPA-29-Pfs48/45-D3 structure are shared among these antibodies, whereas the heavy-chain contacts are more variable (Figures S5A–S5D). Whether a germline-targeting approach for next-generation Pfs48/45 immunogen design can rely on preferentially re-eliciting antibodies with such genetic signatures to enhance the potency of the transmission-blocking response—an approach recently employed for vaccine development in other fields^32^^,^^33^^,^^34^—is an area of future exploration. Importantly, solving the crystal structures of potent human antibodies RUPA-44 and RUPA-117 bound to Pfs48/45-D3 allowed us to describe a potent Pfs48/45 epitope, epitope Ib, in atomic detail. This binding site, like epitope Ia, is highly conserved in *P. falciparum* with only four reported SNPs, none of which individually impacted either RUPA-44 or RUPA-117 binding. Mapping the binding sites of all Pfs48/45 antibodies currently characterized at the molecular level revealed that most potent antibodies against Pfs48/45-D3 bind to one face (Figure 6). This could indicate that in the context of the native parasite, the opposite side of Pfs48/45-D3 may be inaccessible, potentially due to inter-domain contacts within Pfs48/45, interactions with other binding partners, or other *in vivo* considerations. Structural studies of Pfs48/45 in its native context will help evaluate this hypothesis.

Some mAbs against D1 and D3 competed with each other, suggesting that these domains may be in close proximity, which is supported by recent X-ray crystallography structures of full-length Pfs48/45 in the context of mouse-derived antibodies of varying potencies.^28^ An overlay of the co-crystal structures of D3 bound to RUPA-29, RUPA-44, RUPA-117, and RUPA-47 with the full-length crystal structure of Pfs48/45^28^ indicates that these two potent epitopes are accessible in the full-length Pfs48/45 structure, as would be expected (Figure S4E).

In this study, we have only screened the activity of individual human mAbs to Pfs48/45. Future studies may test combinations of mAbs to determine whether mAbs can act synergistically, as previously suggested.^26^ These studies with combinations of mAbs should include RUPA-154, which may target an epitope that spans multiple domains and may be interesting in terms of eliciting additive effectiveness. Furthermore, it will be particularly interesting to determine whether D2-specific mAbs, which appear to be abundant and generally exhibit weak activity, can potentiate mAbs against the other domains or decrease their activity by, for instance, competition. If D2-specific mAbs decrease the activity of mAbs to other domains, this has important implications for the domains that should be included in Pfs48/45 TBV designs. These analyses on mAb potentiation or inhibition should not be restricted to Pfs48/45-specific mAbs but may also include mAbs against other TBV candidates such as Pfs230, as synergy between mAbs against different TBV candidates has been found previously.^35^

In terms of future applicability, the mAbs identified in this study may be valuable for the development of passive and active immunization strategies to reduce malaria transmission. Four mAbs (RUPA-29, -50, -54, and -100) have potencies similar to TB31F, the most potent transmission-blocking mAb described to date. Clinical evaluation of TB31F in humans demonstrated that a single administration can achieve strong TRA for approximately 5 months, an effective time window that would cover peak transmission season in certain Sahelian areas.^13^ The potent mAbs identified in this study may be relevant alternatives or additions to TB31F as they are of human origin and may be less likely to induce anti-drug antibodies. The identification of potent, naturally acquired human mAbs against Pfs48/45 also supports its further development as a TBV. Although clinical evaluation of R0.6C, containing Pfs48/45-D3, is ongoing (Clinicaltrials.gov ID: NCT04862416), our data suggest that the design(s) of next-generation Pfs48/45 vaccines should focus on epitopes Ia and Ib on D3. Future vaccine constructs may also include regions in D1 that are the target of potent mAbs but for which the exact identity still needs to be identified. D2 may be excluded from Pfs48/45-based vaccines as it seemed immunodominant in the Dutch expatriate donor and induced many mAbs with weak potency. The current work thus provides insights into protective and non-protective epitopes that can inform the design of next-generation constructs for this promising TBV antigen and may also form a starting point for effective passive immunization to reduce the transmission of *P. falciparum*.

### Limitations of the study

Our mAbs were obtained from a limited set of two genetically distinct donors with markedly different ages and infection histories. The majority of mAbs, 78, were obtained from a Dutch expatriate who was first exposed to *Plasmodium* at an adult age—only three mAbs were obtained from a Ugandan child who was selected from a large cohort of donors. There were no common germline precursors for mAbs from both donors. Nevertheless, mAbs from more donors would be required to fully capture the diversity of Pfs48/45 responses in different individuals and populations. Earlier studies have indeed suggested that antibody responses to Pfs48/45 differ between individuals.^36^ Furthermore, we used full-length Pfs48/45 produced in insect cells to identify D1- and D2-reactive B cells. Our method using this recombinant glycoprotein (Figure S1C) may, therefore, have failed to identify B cells directed to epitopes that are not glycosylated or differ in glycoforms in native Pfs48/45. Although our structural studies provide molecular detail on the epitopes of functional mAbs against D3, we do not know the exact target epitopes of D1- and D2-specific mAbs nor that of non-functional D3-specific mAbs. Identification of the target epitopes of these mAbs and structural studies on full-length Pfs48/45 with these mAbs will be an important area for future research.

## STAR★Methods

### Key resources table

**Table:** 

REAGENT or RESOURCE	SOURCE	IDENTIFIER
**Antibodies**		

RUPA mAbs (hIgG1)	This paper	N/A
TB31F (Fab)	Kundu et al.^11^	N/A
RUPA-29 (Fab)	This paper	N/A
RUPA-44 (Fab)	This paper	N/A
RUPA-117 (Fab)	This paper	N/A
RUPA-47 (Fab)	This paper	N/A
RUPA-50 (Fab)	This paper	N/A
RUPA-54 (Fab)	This paper	N/A
RUPA-61 (Fab)	This paper	N/A
2544 (IgG1)	McLeod et al.^37^	N/A
399 (IgG1)	This paper	N/A
Alexa Fluor® 488 AffiniPure F(ab’)_2_ fragment goat anti-human IgG, Fcγ fragment specific	Jackson Laboratories	Cat#109-546-098; RRID:AB_2337850
AffiniPure Rabbit Anti-Human IgG, Fcγ fragment specific	Jackson Laboratories	Cat#309-005-008; RRID:AB_2339626
TB31F (IgG1)	Kundu et al.^11^	N/A
anti-Human IgG-HRP	ThermoFisher	Cat# 31412; RRID:AB_228265
anti-human IgG-AF488	Invitrogen	Cat# A-11013; RRID:AB_2534080
85RF45.1	Roeffen et al.^10^	N/A
85RF45.3	Roeffen et al.^10^	N/A
85RF45.5	Roeffen et al.^10^	N/A
Goat Anti-Rat IRDye800	Li-cor	Cat# 926–32219; RRID:AB_1850025
Rabbit Anti-Rat IgG/HRP-conjugated antibody	Dako	Cat# P0450; RRID:AB_2630354
CD19-Brilliant violet 421	Biolegend	Cat# 302233; RRID:AB_10897802
IgG-AlexaFluor 488	Miltenyi Biotec	Cat# 130-093-192; RRID:AB_1036185

**Biological samples**		

PBMCs and plasma from Ugandan donor	Kamya et al.^25^	N/A
PBMCs and plasma from Dutch expatriate	Stone et al.^21^	N/A

**Chemicals, peptides, and recombinant proteins**		

Pfs48/45 6C.mAgE1	McLeod et al.^29^	N/A
Pfs48/45 6C.mAgE1 (K416N; C terminal 6x HIS tag)	This paper	N/A
Pfs48/45 6C.mAgE1 (S322N; C terminal 6x HIS tag)	This paper	N/A
Pfs48/45 6C.mAgE1 (D320H; C terminal 6x HIS tag)	This paper	N/A
Pfs48/45 6C.mAgE1 (L314I; C terminal 6x HIS tag)	This paper	N/A
GIBCO™ FreeStyle™ 293 Expression Medium	Thermo Fisher Scientific	Cat#12338026
FectoPRO DNA Transfection Reagent	VWR	Cat#10118-444
Pfs48/45 R0.6C	Singh et al.^16^	N/A
Pfs48/45 full length	This paper	N/A
Pfs48/45 D1-2 (N28-K289)	This paper	N/A
Pfs48/45 D2-3 (H184-A428)	This paper	N/A
Pfs48/45 D3 (K287-A428)	This paper	N/A
DyLight™ 594 NHS Ester	Thermo Fisher	Cat#46413
DyLight™ 755 NHS Ester	Thermo Fisher	Cat#62279
Pierce IgG elution buffer	Thermo Fisher	Cat#21004
10X HBSTE running buffer	Carterra	Cat#3630
0.1 M MES	Carterra	Cat#3625
10 mM sodium acetate buffer	Carterra	Cat#3801
10 mM glycine pH 2.0	Carterra	Cat#3640
Sulfo–N-hydroxysuccinimide (sulfo-NHS)	Thermo Fisher	Cat#24510
1-ethyl-3-(3-dimethylaminopropyl)carbodiimide (EDC)	Thermo Fisher	Cat#22980

**Critical commercial assays**		

Ni-NTA biosensors	ForteBio	Cat# 18-5102
HC30M chip	Carterra	Cat# 4279
human B Cell Isolation Kit II	Miltenyi Biotec	Cat# 130-091-151
DyLight™ 650 labeling kit	ThermoFisher	Cat# 84535

**Deposited data**		

Crystal structure of Pfs48/45 mAgE1 bound by RUPA-44 and RUPA-29 Fabs	This paper	PDB: 7UXL
Crystal structure of Pfs48/45 mAgE1 bound by RUPA-117 and RUPA-47 Fabs	McLeod et al.^29^	PDB: 7UNB

**Experimental models: Cell lines**		

FreeStyle™ 293-F Cells	Thermo Fisher Scientific	Cat#R79007
*Drosophila melanogaster* S2 cells	Expres2ion Biotechnologies	N/A

**Experimental models: Organisms/strains**		

Parasite: *P. falciparum; NF54 strain*	Ponnudurai et al.^38^	N/A
Mosquito: *Anopheles stephensi* (Nijmegen strain)	Ponnudurai et al.^38^	N/A

**Recombinant DNA**		

pcDNA3.4_RUPA-29 (Fab)	This paper	N/A
pcDNA3.4_RUPA-44 (Fab)	This paper	N/A
pcDNA3.4_RUPA-117 (Fab)	This paper	N/A
pcDNA3.4_RUPA-47 (Fab)	This paper	N/A
pcDNA3.4_RUPA-50 (Fab)	This paper	N/A
pcDNA3.4_RUPA-54 (Fab)	This paper	N/A
pcDNA3.4_RUPA-61 (Fab)	This paper	N/A
pcDNA3.4_TB31F (Fab)	Kundu et al.^11^	N/A
pcDNA3.4_Pfs48/45 6C.mAgE1	McLeod et al.^29^	N/A
pcDNA3.4_Pfs48/45 6C.mAgE1 (K416N; C terminal 6x HIS tag)	This paper	N/A
pcDNA3.4_Pfs48/45 6C.mAgE1 (S322N; C terminal 6x HIS tag)	This paper	N/A
pcDNA3.4_Pfs48/45 6C.mAgE1 (D320H; C terminal 6x HIS tag)	This paper	N/A
pcDNA3.4_Pfs48/45 6C.mAgE1 (L314I; C terminal 6x HIS tag)	This paper	N/A

**Software and algorithms**		

PRISM Graphpad	GraphPad Software, LLC	https://www.graphpad.com/scientific-software/prism/
Phenix	Adams et al.^39^	http://www.phenix-online.org/
Xprep	XPREP^40^	https://www.bruker.com/en/products-and-solutions/diffractometers-and-scattering-systems/single-crystal-x-ray-diffractometers/sc-xrd-software.html
XDS	Kabsch^41^	https://xds.mr.mpg.de/html_doc/downloading.html
Coot	Emsley et al.^42^	https://www2.mrc-lmb.cam.ac.uk/personal/pemsley/coot/
PyMOL	Schrödinger, LLC.	The PyMOL Molecular Graphics System, v2.3.4.
MetaXpress	Molecular Devices	https://www.moleculardevices.com/products/cellular-imaging-systems/acquisition-and-analysis-software/metaxpress
Gen5	Biotek	https://www.biotek.com/products/software-robotics-software/gen5-microplate-reader-and-imager-software/
xPONENT	Luminex	https://www.luminexcorp.com/xponent/#overview
Kinetics analysis software	Carterra	https://carterra-bio.com/applications/kinetics-software/
Epitope analysis software	Carterra	https://carterra-bio.com/applications/epitope-binning-software
R (version 4.1.2)	The R Foundation	https://www.r-project.org/foundation/

**Other**		

QuantumPlex™ optically encoded beads	Bangs Laboratories	Cat#235
Polystyrene beads	Bangs Laboratories	Cat#PC06N

### Resource availability

#### Lead contact

Further information and requests for resources and reagents should be directed to and will be fulfilled by the lead contact, Matthijs M. Jore (matthijs.jore@radboudumc.nl).

#### Materials availability

All unique and stable reagents generated in this study are available via the lead contact upon request.

### Experimental models and subject details

#### Human specimens

Plasma samples were collected from a 69-year-old male Dutch expatriate and from volunteers enrolled in the East African International Centers of Excellence in Malaria Research “PRISM” Tororo study cohort in Tororo, Uganda. This study was conducted between 2013 and 2017; at enrolment 46% of participants were female, the age range was 6 months-68 years and none were symptomatic.^25^ Peripheral blood mononuclear cells (PBMCs) from the Dutch expatriate were isolated in 1994, using a Percoll gradient. For the selected Ugandan donor, an asymptomatic 8-year-old female who at the moment of phlebotomy had an uncomplicated malaria infection with self-reported fever and *a P. falciparum* parasite density of 2080 parasites/uL, PBMCs were isolated from blood collected in acid citrate dextrose (ACD) tubes by Ficoll gradient. Written informed consent was obtained from the parent/guardian of the study participant, and study protocols were approved by the Uganda National Council of Science and Technology (HS 1019), the Makerere University School of Medicine Research and Ethics Committee (Rec No. 2011–167), and the University of California, San Francisco Committee on Human Research (11–05995).

#### Insect cell line culture

For the expression of recombinant Pfs48/45 proteins, *Drosophila melanogaster* S2 cells (ExpreS^2^ion Biotechnologies, Denmark) were transfected according to manufacturer’s instructions to generate stable polyclonal cell lines. These cells were cultured in EX-CELL420 medium (Sigma-Aldrich) at 25°C. The identity of this cell line has not been authenticated; it was used for recombinant expression of proteins of which the identity was confirmed.

#### Human cell line culture

Human cell line HEK 293F (FreeStyle™ 293-F cells, Thermo Fisher Scientific) was cultured in suspension in GIBCO™ FreeStyle™ 293 Expression Medium (Thermo Fisher Scientific) for 6-7 days at 37°C, with 70% humidity and 8% CO_2_ and rotating at 150 RPM. Human cell line HEK 293T was cultured as adherent cells in GIBCO DMEM high glucose medium (Thermo Fisher Scientific) for 6-7 days at 37°C, with 70% humidity and 5% CO_2_. Cell lines were not authenticated as they were used for routine expression of mAbs.

### Method details

#### Donor selection

Gametocyte-specific polyclonal antibody responses in plasma samples from Ugandan donors were assessed by enzyme linked immunosorbent assay (ELISA) as previously described,^43^ using Nunc MaxiSorp™ 96-wells plates (ThermoFisher). Plasma samples were tested at 1:5,400 dilution and considered to have high gametocyte-specific titer when the absorbance (OD450nm) was higher than 1.0. High gametocyte-reactive plasma samples were selected for analysis of Pfs48/45-specific antibody responses by ELISA. Briefly, Nunc MaxiSorp™ 96-wells plates (ThermoFisher) were coated with 1 μg/ml of recombinant full-length Pfs48/45 protein. Plates were blocked with 5% skimmed milk in PBS and subsequently incubated with a 1:200 dilution of plasma. Detection was performed by incubation with 1:60,000 diluted Goat anti-Human IgG/HRP-conjugated antibody (Pierce, Cat. No. 31412). ELISAs were developed using 100 μL tetramethylbenzidine (TMB) and the reaction was stopped with 50 μL 0.2M H_2_SO_4_. Absorbance was measured at 450 nm using an iMark™ Microplate Absorbance Reader (Bio-Rad). The cutoff for seropositivity was defined as the average absorbance of eight malaria-naïve plasma samples from naïve Dutch blood bank donors plus three times the standard deviation. To test functional activity of naturally acquired antibodies in standard membrane feeding assay (SMFA), IgGs were purified from plasma samples using Ab SpinTrap™ columns (Cytiva) following manufacturer’s protocol. Eluted IgGs were buffer-exchanged to milli-Q using Vivaspin® 20 Centrifugal filtration units 30kDa MWCO (Sartorius) and reconstituted in the same volume as the original plasma volume. The IgG concentration was measured on a NanoDrop1000 spectrophotometer (Thermo Scientific) and tested in SMFA (see below)

#### Recombinant Pfs48/45 expression

To isolate and characterize Pfs48/45 specific monoclonal antibodies, recombinant full-length Pfs48/45 was generated. The Pfs48/45 sequence (PF3D7_1346700) was obtained from PlasmoDB.^44^ The glycosylphosphatidylinositol anchor and signal peptide were removed and amino acid residues N28-A428 were codon-optimized for expression in *Drosophila melanogaster* cells (GeneArt, Life Technologies). Predicted N-linked glycosylation sites were left intact. Coding sequences for the different protein fragments were amplified from this codon optimized construct by PCR using Q5® high-fidelity polymerase (New England Biolabs) following manufacturer’s instructions. Pfs48/45 D2-3 comprises amino acid residues H184-A428, Pfs48/45 D1-2 residues N28-K289, and Pfs48/45 D3 residues K287-A428. All constructs were subcloned into a modified pExpreS2-2 plasmid (ExpreS^2^ion Biotechnologies, Denmark) to include a Kozak sequence (GCCACC), an N-terminal His_6_-tag and a BiP insect signal peptide (amino acids KLCILLAVVAFVGLSLG). All plasmids were verified by Sanger sequencing (Baseclear, the Netherlands).

Proteins were produced as previously described.^45^ In short, *Drosophila melanogaster* S2 cells (ExpreS^2^ion Biotechnologies, Denmark) were transfected according to manufacturer’s instructions to generate stable polyclonal cell lines. To capture expressed proteins, clarified five-day batch culture supernatant was filtered and directly applied on a cOmplete™ His-Tag purification column (Sigma-Aldrich). Bound protein was eluted using a gradient elution with imidazole in PBS (pH7.3). Peak fractions were pooled dialyzed at 4°C overnight against PBS using dialysis membrane tubing (Spectra/Por®). Dialyzed samples were loaded on a HiPrep™ 26/60 Sephacryl S-200 HR column (GE Healthcare) connected to an Äkta start system (GE Healthcare). Fractions were analyzed under non-reducing conditions on SDS-PAGE gels stained with Coomassie R-250. Fractions were pooled based on protein quantity and purity.

#### Recombinant Pfs48/45 validation

Proteins were separated on a 4-20% SurePAGE™ Bis-Tris gel (GenScript) alongside a Precision Plus Dual Colour protein marker (Bio-Rad). A final concentration of 10mM dithiothreitol was added to samples that were analyzed under reducing conditions. Total protein was visualized by staining gels directly with InstantBlue™ protein stain (Abcam). Glycosylation of proteins was analyzed by staining gels with the Pierce™ Glycoprotein Staining kit (ThermoScientific). For western blotting, proteins were transferred to nitrocellulose membranes (Bio-Rad) using the TransBlot Turbo System (Bio-Rad). After blocking the membranes with 5% skimmed milk in PBS, rat monoclonal antibodies 85RF45.1, 85RF45.3 and 85RF45.5^10^ were added at 5 μg/ml in PBS with 0.05% Tween in PBS with 0.05% Tween (PBST). The blots were then incubated with 1:10,000 Goat Anti-Rat IRDye800 (Li-cor, Cat. No. 926-32219) and imaged using the Odyssey® CLx system (Li-cor).

In addition, full-length Pfs48/45, D1-2, D2-3 and D3 were validated by ELISA using rat monoclonal antibodies 85RF45.1, 85RF45.3 and 85RF45.5.^10^ Briefly, Nunc MaxiSorp™ 96-wells plates (ThermoFisher) were coated with 1 μg/ml of recombinant protein overnight at 4°C. Plates were blocked with 5% skimmed milk in PBS. The rat monoclonal antibodies were tested in duplicate in a 3-fold serial dilution. Detection was performed with 1:3,000 diluted polyclonal Rabbit Anti-Rat IgG/HRP-conjugated antibody (Cat. No. P0450; Dako, Germany) in 1% skimmed milk/PBST. Plates were developed by adding 100 μl TMB and the reaction was stopped with 50 μL 0.2M H_2_SO_4._ Absorbance was measured at 450 nm using an iMark™ Microplate Absorbance Reader (Bio-Rad).

#### Antigen-specific single B cell sorting and antibody expression

The generation of monoclonal antibodies from antigen-specific single B cells was performed as previously described.^46^ Briefly, PBMCs from the Dutch expatriate were thawed at 37⁰C, counted and resuspended to 2 million cells per ml in RPMI medium supplemented with 10% fetal calf serum (FCS). After 1 h incubation at 37⁰C, B cells were isolated from total PBMCs using the human B Cell Isolation Kit II (MACS, Miltenyi Biotec), following the manufacturer’s instructions. Subsequently, B cells were stained 30 min at 4⁰C with a viability dye (eBioscience™ fixable viability dye eFluor™ 780, Invitrogen). After washing with 2% FCS/PBS (FACS buffer), B cells were incubated for 1 h at 4⁰C with CD19-Brilliant violet 421 (1:25 dilution in FACS buffer; Biolegend, Cat. No. 302233, Clone HIB19), IgG-AlexaFluor 488 (1:10 dilution in FACS buffer; Miltenyi Biotech, Cat. no. 130-093-192, clone IS11-3B2.2.3) and 30 μg/ml full-length Pfs48/45 labelled with the DyLight™ 650 labeling kit (ThermoFisher). Single cell sorting was performed in 96 well plates using an Aria cell sorter (Becton Dickinson). Single cell reverse transcription polymerase chain reaction (RT-PCR) was performed to amplify the gene segments corresponding to the variable region of the heavy (VDJ) and light (VJ) chains of the antibody, using previously validated primers.^46^^,^^47^ The product of the RT-PCR was then sequenced by Sanger sequencing and cloned into expression vectors containing the constant regions of the antibodies for expression as human IgG1. Antibodies were produced by co-transfection of the heavy and light chain expression vectors in a 1:3 ratio with polyethylenimine (Polyscience) to 15-20 million 293T cells.^48^ Supernatants were collected six days post-transfection and antibodies were purified by affinity chromatography with HiTrap Protein A columns (GE Healthcare) on an ÄKTA start system (GE Healthcare).

#### Microfluidics single-cell screening and antibody expression

Screening and recovery of Pfs48/45-specific single B cells using fluorescence microscopy-based microfluidic screening devices was performed as previously described.^49^ Briefly, PBMCs were thawed and memory B cells were differentiated into antibody-secreting cells (ASCs) in culture. ASCs were then injected into microfluidic screening devices containing either 91,000 or 153,000 individual nanoliter-volume reaction chambers. Single cells secreting antibodies specific to full-length Pfs48/45 and the 6C fragment or R0.6C construct^16^ were identified using one of two screening assays. In the multiplexed bead assay, full-length Pfs48/45 and R0.6C were each conjugated to optically encoded beads. Binding of secreted antibodies to the antigen-coupled beads was detected using a fluorescently labeled anti-human IgG secondary antibody. In the soluble antigen assay, secreted antibodies were captured using beads coated with anti-human IgG Fc-specific antibody. Antigen-specific antibodies were identified using soluble Pfs48/45 full-length or 6C fragment, each labelled with a different fluorophore. Positive hits were identified using machine vision and recovered using automated robotics-based protocols.

Next-generation sequencing libraries (MiSeq, Illumina) were produced using single-cell polymerase chain reaction (PCR) and custom molecular biology protocols with automated workstations (Bravo, Agilent). Sequencing data were analyzed using a custom bioinformatics pipeline to yield paired heavy and light chain sequences for recovered antibody-secreting cells.^49^ Each sequence was annotated with the closest germline (V(D)J) genes and degree of somatic hypermutation. Antibodies were considered members of the same clonal family if they shared the same inferred heavy and light V and J genes, and had the same CDR3 length. The variable (V(D)J) region of each antibody chain was synthesized and inserted into expression plasmids and produced as recombinant human IgG1 (GenScript).

#### Domain-specificity of the monoclonal antibodies in ELISA

The domain-specificity of the human monoclonal antibodies was tested in ELISAs with recombinant full-length Pfs48/45 and the three Pfs48/45 fragments. Nunc MaxiSorp™ 96-wells plates (ThermoFisher) were coated with 0.5 μg/ml of recombinant protein overnight at 4°C. Plates were blocked with 5% skimmed milk in PBS before incubation with 10 μg/ml mAb in 1% milk/PBST. Detection was performed by incubation with 1:60,000 Goat anti-Human IgG/HRP-conjugated antibody (Pierce, Cat. No. 31412) in 1% milk/PBST. ELISAs were developed using 100 μl TMB and the reaction was stopped with 50 μL 0.2M H_2_SO_4_. Absorbance was measured at 450 nm using an iMark™ Microplate Absorbance Reader (Bio-Rad). mAbs were considered positive when the absorbance was higher than the mean absorbance plus three standard deviations of seven negative mAbs.

#### Gametocyte western blot

*P. falciparum* NF54 gametocyte extract was prepared as previously described.^43^ The extract was mixed with NuPAGE™ LDS sample buffer and heated for 15 minutes as 56°C. To reduce samples, a final concentration of 25mM dithiothreitol was added. The proteins were separated on a NuPAGE™ 4-12% Bis-Tris 2D-well gel and subsequently transferred to a 0.22 μm nitrocellulose membrane (Bio-Rad) using the Trans-Blot Turbo system (Bio-Rad). The blots were cut into strips, blocked with 5% skimmed milk in PBS and incubated with 5 μg/ml monoclonal antibody. The strips were subsequently incubated with 1:5,000 anti-human IgG-HRP (Pierce, Cat. No 31412) in PBST. Clarity Western ECL substrate (Bio-Rad) was used for development and strips were imaged with the ImageQuant LAS4000 machine (GE Healthcare).

#### Surface immunofluorescence assay

*In vitro* cultured *Plasmodium falciparum* NF54 gametocytes were activated to generate female gametes, which were purified with Nycodenz.^26^ Per condition 10,000 female gametes were incubated with monoclonal antibodies diluted in SIFA buffer (1% heat-inactivated FCS, 0.05% sodium azide in PBS) for 1 hour at 4 ˚C. Samples were washed 3 times with SIFA buffer. Gametes were stained with Hoechst 33342 DNA stain (1:200 dilution) (Invitrogen, cat no. H3570) and anti-human IgG-AF488 (1:400 dilution) (Invitrogen, cat no. A-11013) for 1 hour at 4 ˚C in the dark. Gametes were then washed 3 times with SIFA buffer, fixed with 4% paraformaldehyde and imaged with an ImageXpress Pico automated cell imaging system (Molecular devices). Gametes were then analyzed with MetaXpress software (Molecular devices). Hemozoin and Hoechst-positive gametes were selected and positivity for human antibodies was determined using signal from negative control antibodies as a threshold.

#### Barcoded membrane feeding assay

Barcoded membrane feeding experiments were conducted as previously described.^27^ Briefly, stage V gametocytes from *P. falciparum* luciferase reporter strain NF54-HGL^50^ were mixed with purified mAbs to a final concentration of 100 μg/ml and combined with barcoded *Asaia* bacteria, human red blood cells (50% v/v) human serum (50% v/v) in 96 well plates. The plate was covered with Parafilm and 2-day old *Anopheles stephensi* mosquitoes were fed for 15 minutes. Eight days after feeding, luminescence activity was determined in individual mosquitoes as described previously.^50^ Mosquito homogenates were then pooled in bins for low-infected (>90% reduction in luminescence activity relative to the vehicle controls) and high infected (all other mosquitoes). Barcodes were amplified and quantified from the two bins and the proportion of barcode signals in the low-infected bin was calculated as described previously.^27^

#### Standard membrane feeding assay (SMFA)

SMFA experiments were conducted as previously described and used either *P. falciparum* NF54 wildtype gametocytes with oocyst count readout^38^ or *P. falciparum* NF54-L1 with oocyst expressed luciferase readout,^50^ which can be used interchangeably with in-assay controls. Purified IgGs from naturally exposed donors were tested with wildtype or luciferase expressing parasites, depending on availability. Monoclonal antibodies were tested with wild type parasites and oocysts counts only. Blood meals containing cultured *P. falciparum* gametocytes were fed to *A. stephensi* mosquitoes (Nijmegen colony). All SMFA experiments were conducted in the presence of active complement. For each condition, 20 fully-fed mosquitoes were analyzed. Reported antibody concentrations are concentrations in the total blood meal volume. Transmission reducing activity (TRA) was calculated as the reduction in oocysts compared to a negative control, using a negative binomial regression model as previously described.^45^ SMFA data analyses were done in R (version 4.1.2).

#### Affinity measurements and epitope binning

High-throughput SPR binding and epitope binning experiments were performed on a Carterra LSA instrument equipped with an HC-30M chip type (Carterra-bio) using a 384-ligand array format as described before.^49^ Antibodies at 10 μg/mL or 1 μg/mL in 10 mM NaOAc (pH 4.25) buffer + 0.01% Tween were coupled to the SPR chip using sulfo–N-hydroxysuccinimide (NHS) and 1-ethyl-3-(3-dimethylaminopropyl)carbodiimide (EDC) chemistry.

For binding kinetics and affinity measurements, a threefold dilution series of the antigen of interest, starting at 500 nM in HEPES-buffered saline containing 0.05% Tween 20 and 3 mM EDTA (HBSTE) + 0.05% BSA running buffer, was sequentially injected onto the chip surface with a 5 min association and a 15 min dissociation phase. The chip surface was regenerated using Pierce IgG elution buffer (Thermo Fisher Scientific) + 1 M NaCl for 2 x 60 s in between each dilution series. The data were analyzed using the Carterra Kinetics analysis software.

High-throughput epitope binning experiments were performed in a classical sandwich assay format using HBSTE + 0.05% BSA as running buffer. The antigen at 40 nM was injected for 3 min followed immediately by an antibody analyte at 30 μg/ml for 4 min. The chip surface was regenerated with 10 mM glycine pH 2.0 using 2 x 20 s regeneration cycles. Antigen-only at 40 nM was injected every 8 cycles. The data were analyzed using the Carterra Epitope analysis software for heatmap and competition network generation. Binding responses were normalized to 1 at the end of the antigen binding step. A report time point was set at the end of the antibody analyte step to read out the competition response, relative to the response of the buffer blank analytes at this time point that was nominally set to zero. A threshold was set above this value, such that normalized responses <0.2 were considered blockers and normalized responses >0.3 were considered non-blockers or sandwichers. Normalized responses falling within these limits (0.2-0.3) were considered ambiguous. Antibodies with low coupling to the chip, poor regeneration, or absence of self-blocking were excluded from the binning analysis. Like-behaved antibodies were automatically clustered to form a heatmap and competition plot.

#### Expression and purification of Fabs

Plasmids containing the light and heavy chains of the TB31F, RUPA-44, RUPA-117, RUPA-29, RUPA-47, RUPA-50, RUPA-54, and RUPA-61 Fabs were co-transfected in HEK293F or HEK293S cells depending on the presence of predicted N-linked glycosylation sites. Secreted Fabs were purified using affinity chromatography with either a KappaSelect or LambdaSelect column (GE Healthcare) with 1X PBS and 100 mM glycine pH 2.2 as wash and elution buffers, respectively.^11^ This step was followed by ion-exchange chromatography using a MonoS column (GE Healthcare). 20 mM NaOAc, pH 5.6 with a 0-1 M KCl gradient was used for elution. Fabs expressed in HEK293S cells that contained a putative N-linked glycosylation site were then treated with EndoH.

#### Expression and purification of stabilized Pfs48/45-D3

Stability mutations G397L, H308Y, and I402V were introduced into Pfs48/45-D3 to improve expression yields, purification, monodispersity, and construct stability. Stabilized Pfs48/45-D3 constructs with either L314I, D320H, S322N, K416N, or K414N point mutations were gene synthesized (GeneArt). Pfs48/45-D3 constructs were recombinantly expressed in HEK293S or HEK293F cells, and purified with a HisTrap FF column (GE Healthcare), using TBS, pH 7 with a linear elution gradient of imidazole. Stabilized Pfs48/45-D3 produced in HEK293S cells was then EndoH treated and further purified using size exclusion chromatography with a Superdex 200 Increase column (GE Healthcare) in TBS, pH 7. Throughout the text for the biophysical and structural characterizations, "Pfs48/45-D3” refers to the stabilized construct.

#### Biolayer interferometry (BLI) binding studies

BLI binding experiments were performed using an Octet RED96 instrument. Fabs and purified recombinant Pfs48/45-D3 or Pfs48/45-D3 expression supernatants were diluted in kinetics buffer (PBS, pH 7.4, 0.01% (w/v) BSA, and 0.002% (v/v) Tween-20) as previously described.^11^ Binding kinetics parameters were determined using Ni-NTA biosensors loaded with 10 μg/ml of purified His-tagged Pfs48/45-D3 or expression supernatant followed by a 30 s baseline and an association phase in serially diluted Fab from 500 nM to 15 nM. Biosensors were then dipped into wells containing kinetics buffer for a dissociation step. Epitope binning experiments were done by loading 10 μg/ml of His-tagged Pfs48/45-D3 followed by a 30 s baseline, a 10 min association phase in RUPA-117 or TB31F Fab, and another association phase in the second Fab, as previously described.^37^ Data were analyzed using FortéBio’s Data Analysis software V8.2.

#### Structure determination

Recombinant Pfs48/45-D3, RUPA-29 Fab, and RUPA-44 Fab were complexed at a 1:1.5:1.5 ratio to form a ternary complex and the ternary complex was separated from excess Fab by size exclusion chromatography using a Superdex 200 Increase column (GE Healthcare). The resulting D3-RUPA-44-RUPA-29 sample was concentrated to 6 mg/ml and sitting-drop crystallization experiments were set up with the protein complex and reservoir solution at a 1:1 ratio. D3-RUPA-44-RUPA-29 crystals grew in 0.2 M calcium acetate, 0.1 M MES, pH 6, and 20 % (w/v) polyethylene glycol 8000. Data was collected at the 23ID beamline at the Advanced Photon Source (APS), and subsequently processed using XDS^41^ and Xprep.^40^ Phaser was used for molecular replacement with Pfs48/45-D3 and Fabs from PDB ID’s: 4QF1and 7K8P as search models.^51^ Resulting structures were built and refined using phenix.refine^39^ and Coot^42^ accessed through SBGrid.^52^ Poor electron density for the RUPA-29 Fab constant domain may in part be attributed to two extra “VL” residues introduced aberrantly during cloning in the hinge of the lambda chain. To more accurately account for the experimental density in this region, two conformations of the RUPA-29 constant domain were built with partial occupancies of 0.60 and 0.40.

### Quantification and statistical analysis

Transmission reducing activity (TRA) was calculated as the reduction in oocysts compared to a negative control, using a negative binomial regression model as previously described.^45^ SMFA data analyses were done in R (version 4.1.2).

For binding kinetics measured by surface plasmon resonance, data was analyzed using the Carterra Kinetics analysis software using a 1:1 Langmuir binding model to determine apparent association (k_a_) and dissociation (k_d_) kinetic rate constants and apparent binding affinity constants (K_D_). For binding kinetics calculated by biolayer interferometry, data was analyzed using the Octet software (Sartorius ForteBio, version 8.2.0.7) according to the manufacturer’s instructions.

Statistical analyses were done in Graphpad PRISM and details about the analyses can be found in the figure legend. p < 0.05 was considered significant.

## Data Availability

• Antibody sequences are available in Table S7. The crystal structure has been deposited in the Protein Data Bank and is publicly available as of the date of publication. Accession number is listed in the key resources table. • This paper does not report original code. • Any additional information required to reanalyze the data reported in this paper is available from the lead contact upon request.

## Supplemental information

Document S1. Figures S1–S5, Tables S1–S6, and supplemental reference

Table S7. Raw standard membrane-feeding assay (SMFA) data and monoclonal antibody sequences, related to Figures 1 and 2 and STAR Methods

Document S2. Article plus supplemental information
