# Supplementary material for: Highly potent, naturally acquired human monoclonal antibodies against Pfs48/45 block Plasmodium falciparum transmission to mosquitoes
Source: Immunity. 2023 Feb 14;56(2):406–419.e7. doi: 10.1016/j.immuni.2023.01.009 (PMC9942873; doi:10.1016/j.immuni.2023.01.009)
Supplement: Document S1. Figures S1–S5, Tables S1–S6, and supplemental reference [file mmc1.pdf]

Immunity, Volume *56*

Supplemental information

Highly potent, naturally acquired human monoclonal antibodies against Pfs48/45 block *Plasmodium falciparum* transmission to mosquitoes

Amanda Fabra-García, Sophia Hailemariam, Roos M. de Jong, Kirsten Janssen, Karina Teelen, Marga van de Vegte-Bolmer, Geert-Jan van Gemert, Danton Ivanochko, Anthony Semesi, Brandon McLeod, Martijn W. Vos, Marloes H.C. de Bruijni, Judith M. Bolscher, Marta Szabat, Stefanie Vogt, Lucas Kraft, Sherie Duncan, Moses R. Kamya, Margaret E. Feeney, Prasanna Jagannathan, Bryan Greenhouse, Koen J. Dechering, Robert W. Sauerwein, C. Richter King, Randall S. MacGill, Teun Bousema, Jean-Philippe Julien, and Matthijs M. Jore

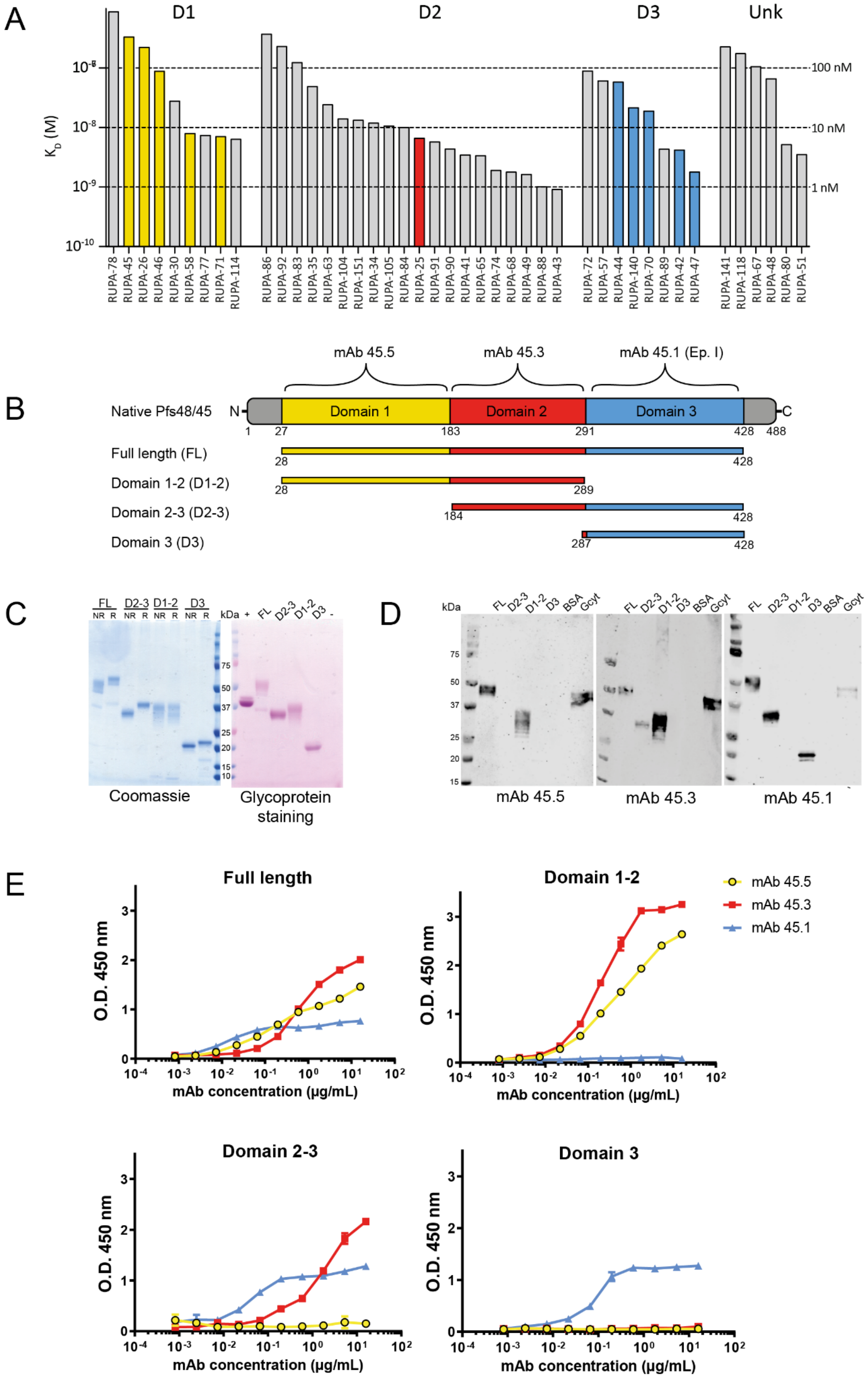
A
D1
D2
D3
Unk
KD (M)
100 nM
10 nM
1 nM
RUPA-78 RUPA-45 RUPA-26 RUPA-46 RUPA-30 RUPA-58 RUPA-77 RUPA-71 RUPA-114
RUPA-86 RUPA-92 RUPA-83 RUPA-35 RUPA-63 RUPA-104 RUPA-151 RUPA-34 RUPA-105 RUPA-84 RUPA-25 RUPA-91 RUPA-90 RUPA-41 RUPA-65 RUPA-74 RUPA-68 RUPA-49 RUPA-88 RUPA-43
RUPA-72 RUPA-57 RUPA-44 RUPA-140 RUPA-70 RUPA-89 RUPA-42 RUPA-47
RUPA-141 RUPA-118 RUPA-67 RUPA-48 RUPA-80 RUPA-51
B
mAb 45.5
mAb 45.3
mAb 45.1 (Ep. I)
Native Pfs48/45
Domain 1
Domain 2
Domain 3
Full length (FL)
Domain 1-2 (D1-2)
Domain 2-3 (D2-3)
Domain 3 (D3)
C
Coomassie
Glycoprotein staining
D
mAb 45.5
mAb 45.3
mAb 45.1
E
Full length
Domain 1-2
Domain 2-3
Domain 3
mAb 45.5
mAb 45.3
mAb 45.1
O.D. 450 nm
mAb concentration (µg/mL)

Figure S1. Binding of mAbs to recombinant Pfs48/45. (**a**) Binding affinity (K_D) of mAbs for Pfs48/45. Affinity was determined by surface plasmon resonance (SPR) with immobilized antibodies and full-length Pfs48/45 as analyte. mAbs are grouped according to domain specificity. Colored bars represent mAbs with >80% TRA at 100 µg/ml. (**b-e**) Expression and validation of recombinant Pfs48/45 fragments. (**b**) Schematic overview of Pfs48/45 fragments produced in Drosophila melanogaster S2 cells, including amino acid numbers. Rodent antibodies 85RF45.5, 85RF45.3 and 85RF45.1 recognize D1, D2 and D3, respectively[1]. mAbs 85RF45.3 and 85RF45.1 recognize conformational epitopes, 85RF45.5 recognizes a linear epitope[1]. Ep. I, Epitope I. (**c**) Purified proteins were separated on a 4-20% Bis-Tris polyacrylamide gel under non-reducing (NR) and reducing (R) conditions and were visualized with coomassie staining (left) or Pierce® glycoprotein staining (right). (+) and (-) are positive and negative glycosylation controls, respectively. The presence of (conformational) epitopes in recombinant fragments was confirmed by western blot (**d**) and ELISA (**e**). Gcyt, gametocyte extract. BSA, bovine serum albumin. **Related to Figure 1.**

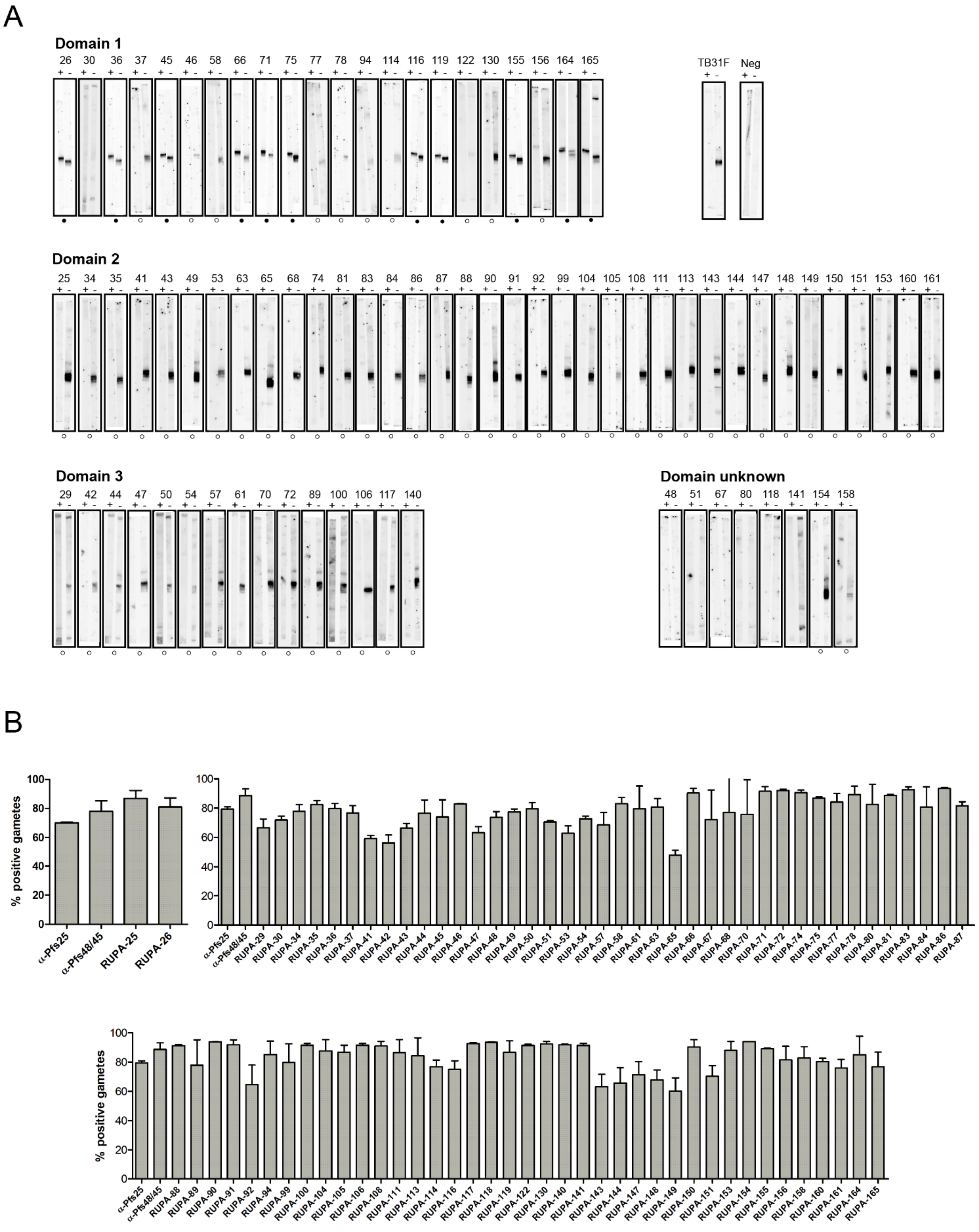

Figure S2. Recognition of native Pfs48/45 by RUPA antibodies. (**a**) Binding of mAbs to gametocyte extract. Gametocyte extract was run on SDS-PAGE gel under reducing (+) and non-reducing (-) conditions. After transfer to nitrocellulose membrane, strips were cut and incubated with individual RUPA antibodies. TB31F was included as a positive control antibody that is conformation-dependent. A non-targeting human IgG was included as negative control (Neg). Closed circles indicate mAbs that

bind to linear epitopes, while open circles indicate mAbs that bind to conformational epitopes. (**b**) Female gamete recognition by mAbs in surface immunofluorescence assay. All antibodies were tested at 5 µg/mL, except for mAbs against Pfs25 (2544, 0.1 µg/mL), Pfs48/45 (TB31F, 0.03 µg/mL). RUPA-25 and RUPA-26 were tested at 5 µg/mL in a separate experiment that included mAbs against Pfs25 (2544, 1 µg/mL) and Pfs48/45 (TB31F, 0.1 µg/mL). Anti-PfCSP mAb 399, which is sporozoite specific and does not recognize female gametes, was used for background correction. Values represent means of two technical replicates after background correction, and error bars show s.d. **Related to Figure 1.**

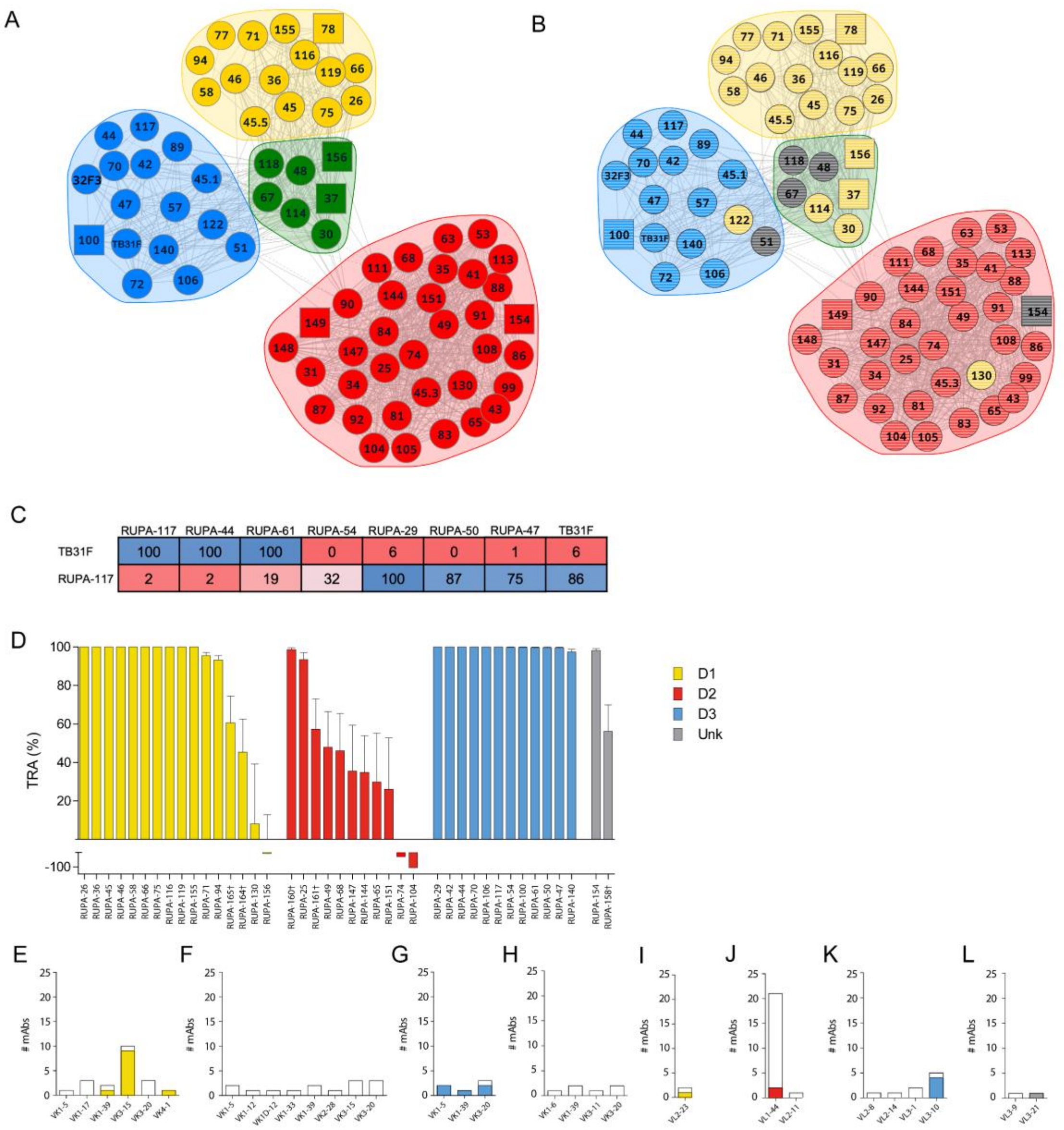

Figure S3. Competition experiments identified four groups of monoclonal antibodies, transmission reducing activity (TRA) of potent monoclonal antibodies (mAbs) in standard membrane feeding assay (SMFA) and genetic characteristics of light chains. (**a**) Competition between monoclonal antibodies (mAbs) was tested in two orientations; one antibody coupled to a chip and the other one in solution, and vice versa. mAbs with data in two directions are shown in circles and those with data in one direction as squares. (**b**) As in a, but with individual mAbs colored following domain specificity as determined by construct ELISA (Table S1). Yellow, D1; red, D2; blue, D3 and grey, unknown domain. (**c**) Epitope binning of anti-Pfs48/45-D3 Fabs using biolayer interferometry**.** Binning data is shown as a matrix where numbers indicate the percentage of potential binding. High competition is displayed in red (low numbers) and low competition is displayed in blue (high numbers). (**d**) 36 mAbs that showed the highest potency in barcoded membrane feeding assay (Figure 2a) and five mAbs that were not available at the time of high throughput membrane feeding assay (marked with †) were tested at 100 µg/ml in SMFA with *Anopheles stephensi* mosquitoes and *Plasmodium falciparum* NF54 gametocytes. TRA was calculated as the percentage reduction in oocyst intensity compared to a non-antibody control and obtained from a single feeding experiment with 20 mosquitoes per condition. Error bars indicate 95% confidence intervals. Raw SMFA data is presented in Supplementary file 1. (**e-l**) Genetic characteristics of light chains. Bar graphs showing number of monoclonal antibodies (mAbs) per (**e-h**)

kappa and (**i-l**) lambda light chain family. mAbs are grouped by (**e,i**) D1-, (**f,j**) D2- and (**g,k**) D3-specific mAbs and (**h,l**) mAbs with unknown specificity. Colored bars represent mAbs with >80% transmission reducing activity at 100 µg/ml. **Related to Figures 1, 2 and 3.**

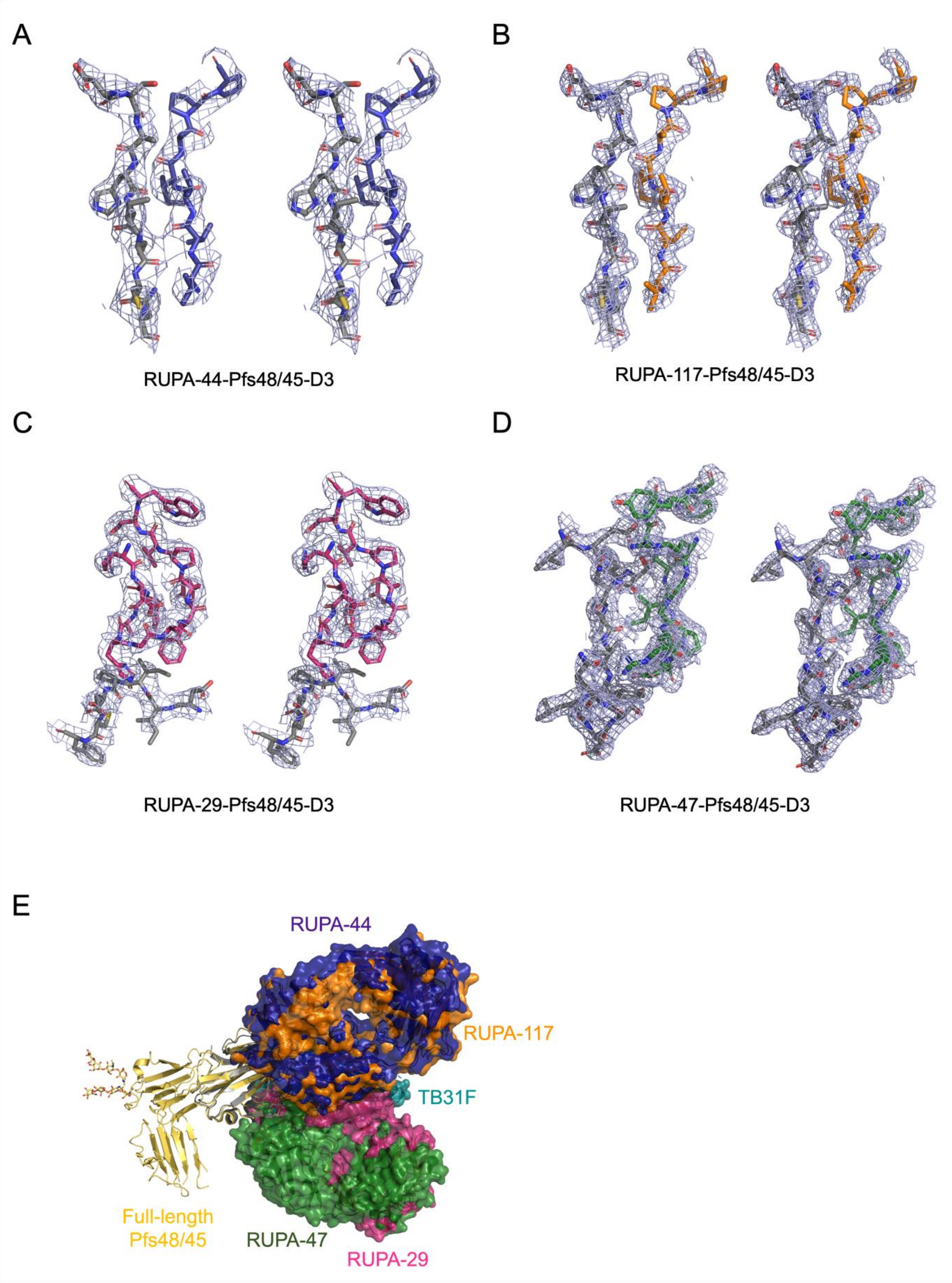

Figure S4. Protective epitopes of potent human antibodies are accessible in full-length Pfs48/45 structure. (**a**) Stereo view of the 2mFo-DFc composite omit electron density map contoured at 1.5 σ. Structures of interfaces between Pfs48/45-D3 in grey and RUPA-44 (**a**) in blue, RUPA-117 (**b**) in orange, RUPA-29 (**c**) in pink, and RUPA-47 (**d**) in green. Electron density is shown as a blue mesh. (**e**) Fabs RUPA-

44 (dark blue), RUPA-117 (orange), TB31F (teal; PDB ID: 6E63), RUPA-29 (pink), and RUPA-47 (dark green) bound to D3 (grey) overlayed onto full-length Pfs48/45 (yellow; PDB:7ZXF). **Related to Figures 4 and 5.**

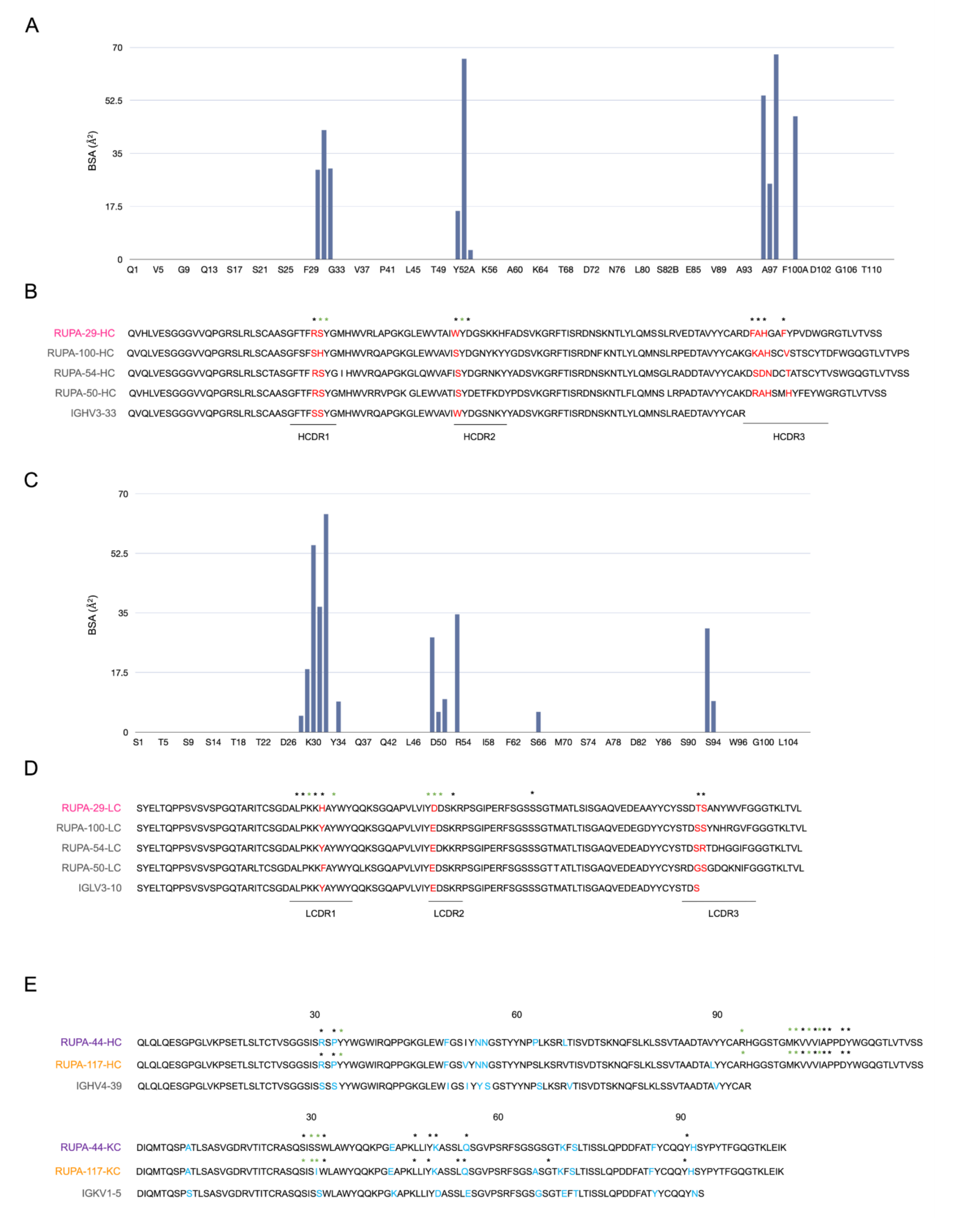

Figure S5. Alignment of antibodies from potent antibody lineages targeting Pfs48/45-D3 epitopes Ia and Ib. (**a**) Buried surface area of each RUPA-29-HC residue in the Pfs48/45-D3-RUPA-29 complex. (**b**) Alignment of the heavy chain sequences of RUPA-29, RUPA-100, RUPA-54, RUPA-50, and germline precursor, IGHV3-33. (**c**) Buried surface area of each RUPA-29-LC residue in the Pfs48/45-D3-RUPA-29 complex. (**d**) Alignment of the lambda chain sequences of RUPA-29, RUPA-100, RUPA-54, RUPA-50, and germline precursor, IGLV3-10. Green asterisks indicate RUPA-29 residues that form H-bonds/salt-bridges, while black asterisks indicate residues that make van der Waals (VdW) interactions with Pfs48/45-D3. Amino acids in red signify contact residues that are not conserved across all antibodies.

(**e**) Alignment of heavy and light variable chain sequences of RUPA-44 and RUPA-117 with their inferred germline precursor. Mutated residues from the germline are in blue. Asterisks indicate residues that form VdW (black) or electrostatic (green) contacts with Pfs48/45-D3. **Related to Figures 4 and 5.**

Table S1. Domain specificity of isolated monoclonal antibodies (mAbs) as determined by ELISA. mAbs that were positive against full-length Pfs48/45, were also tested against fragments of Pfs48/45 (Supplementary figure 3). mAbs were considered Domain 1-specific mAbs if they were positive for Domain 1-2 and negative for Domain 2-3 and Domain 3, Domain 2-specific mAbs if positive for Domain 1-2 and Domain 2-3 and negative for Domain 3, and Domain 3-specific mAbs if positive for Domain 2-3 and Domain 3 and negative for Domain 1-2. Other mAbs have unknown (unk) specificity. Optical density (OD_{450}) values are means of three technical replicates and the standard deviation (SD) is shown between brackets. The cutoff was determined by calculating the mean + three standard deviations of seven non-Pfs48/45 mAbs that were included on each ELISA plate. Two mAbs, marked with an asterisk, were negative in full-length Pfs48/45 ELISA but showed high affinity for full-length Pfs48/45 by surface plasmon resonance and were therefore included in further analyses. **Related to Figure 1.**

Name	Full-length			Domain 1-2			Domain 2-3			Domain 3			Domain specificity
	OD_{450} (SD)	Cutoff	Pos=1/ Neg=0	OD_{450} (SD)	Cutoff	Pos=1/ Neg=0	OD_{450} (SD)	Cutoff	Pos=1/ Neg=0	OD_{450} (SD)	Cutoff	Pos=1/ Neg=0	
**RUPA-25**	1.38 (0.51)	0.14	1	2.13 (0.04)	0.30	1	1.62 (0.09)	0.19	1	0.05 (0.01)	0.15	0	**2**
**RUPA-26**	1.28 (0.06)	0.14	1	1.54 (0.07)	0.30	1	0.07 (0.02)	0.19	0	0.06 (0.01)	0.15	0	**1**
**RUPA-29**	1.60 (0.04)	0.14	1	0.15 (0.00)	0.30	0	1.69 (0.14)	0.19	1	1.84 (0.04)	0.15	1	**3**
**RUPA-30**	0.66 (0.72)	0.14	1	0.35 (0.03)	0.30	1	0.15 (0.02)	0.19	0	0.10 (0.03)	0.15	0	**1**
**RUPA-34**	1.30 (0.27)	0.14	1	1.85 (0.05)	0.30	1	0.35 (0.02)	0.19	1	0.05 (0.01)	0.15	0	**2**
**RUPA-35**	1.33 (0.08)	0.14	1	1.93 (0.03)	0.30	1	1.15 (0.08)	0.19	1	0.13 (0.03)	0.15	0	**2**
**RUPA-36**	1.09 (0.04)	0.14	1	1.64 (0.06)	0.30	1	0.05 (0.00)	0.19	0	0.03 (0.00)	0.15	0	**1**
**RUPA-37**	0.80 (0.05)	0.14	1	0.79 (0.04)	0.30	1	0.06 (0.00)	0.19	0	0.04 (0.00)	0.15	0	**1**
**RUPA-41**	1.58 (0.11)	0.14	1	2.17 (0.09)	0.30	1	1.54 (0.06)	0.19	1	0.05 (0.00)	0.15	0	**2**
**RUPA-42**	1.22 (0.05)	0.14	1	0.12 (0.00)	0.30	0	1.36 (0.06)	0.19	1	1.55 (0.11)	0.15	1	**3**
**RUPA-43**	1.50 (0.33)	0.14	1	2.20 (0.02)	0.30	1	1.71 (0.07)	0.19	1	0.06 (0.00)	0.15	0	**2**
**RUPA-44**	1.81 (0.11)	0.14	1	0.29 (0.01)	0.30	0	1.80 (0.06)	0.19	1	1.81 (0.11)	0.15	1	**3**
**RUPA-45**	0.94 (0.20)	0.14	1	1.20 (0.03)	0.30	1	0.11 (0.00)	0.19	0	0.05 (0.01)	0.15	0	**1**
**RUPA-46**	0.61 (0.04)	0.14	1	0.56 (0.02)	0.30	1	0.10 (0.01)	0.19	0	0.04 (0.00)	0.15	0	**1**
**RUPA-47**	1.67 (0.04)	0.14	1	0.23 (0.01)	0.30	0	1.84 (0.07)	0.19	1	1.81 (0.05)	0.15	1	**3**
**RUPA-48**	0.39 (0.02)	0.14	1	0.17 (0.01)	0.30	0	0.07 (0.01)	0.19	0	0.05 (0.01)	0.15	0	**Unk**
**RUPA-49**	1.54 (0.07)	0.14	1	2.34 (0.14)	0.30	1	1.69 (0.03)	0.19	1	0.06 (0.01)	0.15	0	**2**
**RUPA-50**	1.49 (0.05)	0.14	1	0.14 (0.00)	0.30	0	1.68 (0.03)	0.19	1	1.70 (0.05)	0.15	1	**3**
**RUPA-51**	0.46 (0.04)	0.14	1	0.10 (0.01)	0.30	0	0.08 (0.01)	0.19	0	0.08 (0.06)	0.15	0	**Unk**
**RUPA-53**	1.86 (0.06)	0.14	1	2.45 (0.15)	0.30	1	1.84 (0.03)	0.19	1	0.06 (0.02)	0.15	0	**2**
**RUPA-54**	1.61 (0.10)	0.18	1	0.23 (0.12)	0.26	0	1.87 (0.02)	0.19	1	1.97 (0.04)	0.23	1	**3**
**RUPA-57**	1.44 (0.04)	0.18	1	0.08 (0.01)	0.26	0	1.71 (0.02)	0.19	1	1.75 (0.01)	0.23	1	**3**
**RUPA-58**	1.29 (0.04)	0.18	1	0.97 (0.04)	0.26	1	0.12 (0.03)	0.19	0	0.19 (0.01)	0.23	0	**1**
**RUPA-59**	0.09 (0.01)	0.18	0										
**RUPA-61**	1.65 (0.06)	0.18	1	0.10 (0.01)	0.26	0	1.78 (0.03)	0.19	1	1.89 (0.15)	0.23	1	**3**
**RUPA-63**	1.68 (0.04)	0.18	1	2.14 (0.05)	0.26	1	1.57 (0.00)	0.19	1	0.05 (0.00)	0.23	0	**2**
**RUPA-65**	2.05 (0.03)	0.18	1	2.16 (0.15)	0.26	1	1.75 (0.08)	0.19	1	0.19 (0.02)	0.23	0	**2**
**RUPA-66**	1.39 (0.02)	0.18	1	1.50 (0.09)	0.26	1	0.10 (0.04)	0.19	0	0.19 (0.01)	0.23	0	**1**
**RUPA-67**	0.23 (0.01)	0.18	1	0.06 (0.00)	0.26	0	0.11 (0.04)	0.19	0	0.17 (0.06)	0.23	0	**Unk**
**RUPA-68**	1.86 (0.03)	0.18	1	2.02 (0.11)	0.26	1	1.53 (0.05)	0.19	1	0.17 (0.07)	0.23	0	**2**
**RUPA-70**	1.76 (0.06)	0.18	1	0.16 (0.00)	0.26	0	1.66 (0.07)	0.19	1	2.23 (0.07)	0.23	1	**3**
**RUPA-71**	1.39 (0.02)	0.18	1	1.02 (0.20)	0.26	1	0.13 (0.02)	0.19	0	0.22 (0.01)	0.23	0	**1**
**RUPA-72**	1.30 (0.04)	0.18	1	0.06 (0.01)	0.26	0	1.37 (0.03)	0.19	1	1.56 (0.04)	0.23	1	**3**
**RUPA-74**	1.96 (0.09)	0.18	1	2.32 (0.11)	0.26	1	1.71 (0.06)	0.19	1	0.09 (0.03)	0.23	0	**2**
**RUPA-75**	1.37 (0.06)	0.18	1	1.54 (0.13)	0.26	1	0.10 (0.05)	0.19	0	0.10 (0.07)	0.23	0	**1**
**RUPA-77**	1.24 (0.08)	0.18	1	0.97 (0.04)	0.26	1	0.06 (0.00)	0.19	0	0.07 (0.01)	0.23	0	**1**
**RUPA-78**	0.89 (0.03)	0.18	1	0.65 (0.08)	0.26	1	0.05 (0.00)	0.19	0	0.06 (0.01)	0.23	0	**1**
**RUPA-80***	0.08 (0.00)	0.18	0										**Unk**
**RUPA-81**	1.70 (0.12)	0.18	1	1.93 (0.05)	0.26	1	0.70 (0.04)	0.19	1	0.16 (0.07)	0.23	0	**2**
**RUPA-83**	1.79 (0.07)	0.18	1	2.02 (0.11)	0.26	1	1.46 (0.01)	0.19	1	0.15 (0.08)	0.23	0	**2**
**RUPA-84**	1.54 (0.07)	0.14	1	2.07 (0.07)	0.27	1	1.63 (0.03)	0.15	1	0.08 (0.01)	0.25	0	**2**
**RUPA-86**	1.32 (0.15)	0.14	1	1.84 (0.02)	0.27	1	1.34 (0.05)	0.15	1	0.08 (0.00)	0.25	0	**2**

**RUPA-87**	1.99 (0.15)	0.14	1	2.15 (0.23)	0.27	1	1.81 (0.09)	0.15	1	0.10 (0.01)	0.25	0	**2**
**RUPA-88**	1.88 (0.09)	0.14	1	2.07 (0.14)	0.27	1	1.71 (0.10)	0.15	1	0.09 (0.00)	0.25	0	**2**
**RUPA-89**	1.53 (0.05)	0.14	1	0.13 (0.01)	0.27	0	1.54 (0.01)	0.15	1	1.76 (0.01)	0.25	1	**3**
**RUPA-90**	1.64 (0.15)	0.14	1	2.14 (0.06)	0.27	1	1.75 (0.13)	0.15	1	0.08 (0.01)	0.25	0	**2**
**RUPA-91**	1.45 (0.06)	0.14	1	2.05 (0.14)	0.27	1	1.48 (0.06)	0.15	1	0.06 (0.00)	0.25	0	**2**
**RUPA-92**	0.95 (0.03)	0.14	1	1.57 (0.07)	0.27	1	0.79 (0.09)	0.15	1	0.07 (0.01)	0.25	0	**2**
**RUPA-94**	0.82 (0.06)	0.14	1	0.57 (0.07)	0.27	1	0.06 (0.00)	0.15	0	0.07 (0.01)	0.25	0	**1**
**RUPA-99**	1.70 (0.08)	0.14	1	2.06 (0.19)	0.27	1	1.75 (0.03)	0.15	1	0.08 (0.00)	0.25	0	**2**
**RUPA-100**	1.54 (0.08)	0.14	1	0.14 (0.01)	0.27	0	1.77 (0.05)	0.15	1	1.92 (0.05)	0.25	1	**3**
**RUPA-102**	0.05 (0.00)	0.14	0										
**RUPA-104**	1.96 (0.08)	0.14	1	2.21 (0.16)	0.27	1	1.81 (0.06)	0.15	1	0.08 (0.01)	0.25	0	**2**
**RUPA-105**	1.55 (0.08)	0.14	1	1.90 (0.14)	0.27	1	1.51 (0.04)	0.15	1	0.07 (0.00)	0.25	0	**2**
**RUPA-106**	0.97 (0.09)	0.14	1	0.06 (0.02)	0.27	0	1.14 (0.06)	0.15	1	1.25 (0.08)	0.25	1	**3**
**RUPA-107**	0.06 (0.00)	0.14	0										
**RUPA-108**	1.25 (0.10)	0.14	1	1.73 (0.10)	0.27	1	0.35 (0.05)	0.15	1	0.07 (0.01)	0.25	0	**2**
**RUPA-110**	0.05 (0.01)	0.14	0										
**RUPA-111**	1.86 (0.17)	0.14	1	2.23 (0.21)	0.27	1	1.94 (0.08)	0.15	1	0.09 (0.03)	0.25	0	**2**
**RUPA-113**	1.59 (0.05)	0.14	1	2.01 (0.08)	0.27	1	1.62 (0.05)	0.15	1	0.07 (0.00)	0.25	0	**2**
**RUPA-114**	0.67 (0.05)	0.21	1	0.45 (0.05)	0.30	1	0.14 (0.01)	0.22	0	0.19 (0.01)	0.31	0	**1**
**RUPA-116**	1.16 (0.07)	0.21	1	1.43 (0.06)	0.30	1	0.03 (0.00)	0.12	0	0.17 (0.01)	0.31	0	**1**
**RUPA-117**	1.65 (0.06)	0.21	1	0.24 (0.02)	0.30	0	2.07 (0.09)	0.22	1	2.07 (0.11)	0.31	1	**3**
**RUPA-118***	0.12 (0.01)	0.21	0										**Unk**
**RUPA-119**	1.23 (0.09)	0.21	1	1.13 (0.04)	0.30	1	0.05 (0.00)	0.22	0	0.19 (0.04)	0.31	0	**1**
**RUPA-120**	0.06 (0.00)	0.21	0										
**RUPA-121**	0.11 (0.11)	0.21	0										
**RUPA-122**	0.82 (0.03)	0.21	1	0.35 (0.03)	0.30	1	0.11 (0.03)	0.22	0	0.18 (0.01)	0.31	0	**1**
**RUPA-124**	0.05 (0.00)	0.21	0										
**RUPA-125**	0.05 (0.01)	0.21	0										
**RUPA-127**	0.06 (0.01)	0.21	0										
**RUPA-130**	1.06 (0.03)	0.21	1	1.40 (0.10)	0.30	1	0.11 (0.00)	0.22	0	0.07 (0.05)	0.31	0	**1**
**RUPA-132**	0.06 (0.01)	0.21	0										
**RUPA-140**	1.54 (0.02)	0.21	1	0.11 (0.01)	0.30	0	1.78 (0.09)	0.22	1	1.65 (0.10)	0.31	1	**3**
**RUPA-141**	0.26 (0.07)	0.21	1	0.08 (0.01)	0.30	0	0.15 (0.01)	0.22	0	0.07 (0.01)	0.31	0	**Unk**
**RUPA-142**	0.06 (0.00)	0.21	0										
**RUPA-143**	0.37 (0.06)	0.21	1	0.42 (0.04)	0.30	1	0.32 (0.03)	0.22	1	0.06 (0.03)	0.31	0	**2**
**RUPA-144**	1.54 (0.04)	0.21	1	1.99 (0.07)	0.30	1	1.58 (0.09)	0.22	1	0.14 (0.08)	0.31	0	**2**
**RUPA-145**	0.15 (0.09)	0.21	0										
**RUPA-146**	0.06 (0.00)	0.25	0										
**RUPA-147**	1.82 (0.04)	0.25	1	2.08 (0.06)	0.22	1	1.62 (0.03)	0.10	1	0.05 (0.00)	0.13	0	**2**
**RUPA-148**	1.78 (0.05)	0.25	1	2.14 (0.04)	0.22	1	1.66 (0.02)	0.10	1	0.05 (0.00)	0.13	0	**2**
**RUPA-149**	1.01 (0.04)	0.25	1	1.21 (0.22)	0.22	1	0.75 (0.15)	0.10	1	0.05 (0.00)	0.13	0	**2**
**RUPA-150**	0.71 (0.03)	0.25	1	0.78 (0.17)	0.22	1	0.45 (0.08)	0.10	1	0.04 (0.00)	0.13	0	**2**
**RUPA-151**	1.73 (0.05)	0.25	1	2.15 (0.06)	0.22	1	1.53 (0.03)	0.10	1	0.05 (0.01)	0.13	0	**2**
**RUPA-152**	0.22 (0.01)	0.25	0										
**RUPA-153**	0.51 (0.03)	0.25	1	0.85 (0.08)	0.22	1	0.34 (0.04)	0.10	1	0.09 (0.00)	0.13	0	**2**
**RUPA-154**	1.67 (0.10)	0.25	1	1.99 (0.05)	0.22	1	1.69 (0.01)	0.10	1	0.22 (0.03)	0.13	1	**Unk**
**RUPA-155**	1.24 (0.14)	0.25	1	1.48 (0.20)	0.22	1	0.07 (0.00)	0.10	0	0.05 (0.00)	0.13	0	**1**
**RUPA-156**	0.50 (0.09)	0.25	1	0.49 (0.02)	0.22	1	0.08 (0.01)	0.10	0	0.05 (0.00)	0.13	0	**1**
**RUPA-157**	0.06 (0.00)	0.25	0										
**RUPA-158**	0.38 (0.05)	0.25	1	0.15 (0.01)	0.22	0	0.07 (0.00)	0.10	0	0.04 (0.00)	0.13	0	**Unk**
**RUPA-159**	0.09 (0.01)	0.25	0										
**RUPA-160**	1.33 (0.09)	0.25	1	1.63 (0.15)	0.22	1	1.01 (0.03)	0.10	1	0.04 (0.00)	0.13	0	**2**
**RUPA-161**	1.76 (0.02)	0.25	1	2.10 (0.06)	0.22	1	1.72 (0.10)	0.10	1	0.05 (0.00)	0.13	0	**2**
**RUPA-162**	0.07 (0.01)	0.25	0										
**RUPA-163**	0.12 (0.03)	0.25	0										
**RUPA-164**	0.32 (0.04)	0.25	1	0.58 (0.06)	0.22	1	0.05 (0.00)	0.10	0	0.04 (0.00)	0.13	0	**1**
**RUPA-165**	0.85 (0.10)	0.25	1	1.12 (0.09)	0.22	1	0.05 (0.00)	0.10	0	0.04 (0.00)	0.13	0	**1**

Table S2. Gene characteristics of Pfs48/45-specific monoclonal antibodies (mAbs). mAbs are grouped by donor (D=Dutch expatriate, U=Ugandan donor) and domain specificity. **Related to Figure 3.**

Donor	Domain	mAb	V_H	V_H Identity to germline (%)	D_H	J_H	HCDR3 (AA)	V_L	V_L Identity to germline (%)	J_L	LCDR3 (AA)
D	1	RUPA-26	VH4-34	90.4	DH6-19	JH5	ARRSSGYYRGSRYTGWFDP	VK3-15	96.1	JK4	QQYNNWPGLT
		RUPA-30	VH1-46	92.2	DH2-15	JH3	ARAGEYCRGIRCYIDGLDI	VK1-17	97.9	JK1	LQHNDSPWT
		RUPA-36	VH4-34	89.0	DH6-19	JH5	ARRASGWYRQSRYTGWFDP	VK3-15	98.2	JK4	QQYNNWPGLS
		RUPA-37	VH1-46	88.5	DH2-15	JH3	ARAGEYCRGIRCYVDGIDM	VK1-17	97.5	JK1	LQHNSSPWT
		RUPA-45	VH4-34	89.0	DH6-19	JH5	ARRSSGYYRGSHYTGWFDP	VK3-15	96.1	JK4	QQYNNWPGLT
		RUPA-46	VH3-11	92.6	DH2 or 15-2a	JH3	ARDRKGRNSETYYGALDV	VK4-1	95.4	JK1	QQYYTTPTWT
		RUPA-58	VH1-2	94.6	DH2-2	JH4	ARDYCTGSSCYRTDYDY	VK1-39	94.0	JK5	QQSYRDPIT
		RUPA-66	VH4-34	96.2	DH6-19	JH5	ARRSSGWYRRSRYTGWFDP	VK3-15	96.5	JK4	QQYNNWPGLS
		RUPA-71	VH3-49	90.3	DH3-22	JH3	TRNGLRWYDSSGPRGWAFDI	VK3-15	97.6	JK1	QQYNNWPPWT
		RUPA-75	VH4-34	91.8	DH3-22	JH5	ARRSSGYYRGSRYTGWLDP	VK3-15	96.1	JK4	QQYNNWPGLT
		RUPA-77	VH2-5	92.3	DH6-6	JH5	AHRILAAYNYFDP	VK1-39	93.3	JK4	QQSYTSPLT
		RUPA-78	VH3-23	90.1	DH6-6	JH3	AKFRTPRFSSSSGDALDL	VK3-20	96.5	JK2	QQYGVSPGT
		RUPA-94	VH4-39	92.3	DH3-16	JH4	ARRPNPGFWGGGYYFDY	VL2-23	98.0	JL1	CSYADSSTRNYV
		RUPA-114	VH1-18	92.4	DH5-12	JH6	ARGGDRGDGLRFYYFALDV	VK3-20	96.5	JK1	QLYGRSSWT
		RUPA-116	VH4-34	94.8	DH6-19	JH5	ARRSSGYYRQSRYTGWFDP	VK3-15	96.8	JK4	QQYNNWPGLT
		RUPA-119	VH4-34	89.0	DH6-19	JH5	ARRSSGWYRQSRYTGWFDP	VK3-15	98.2	JK4	QQYNNWPGLT
		RUPA-122	VH2-5	91.3	DH3-10	JH5	ARTMKKITMIRGVPYEGGVQGFDP	VK1-17	94.7	JK4	QQYSGYFT
		RUPA-155	VH4-34	90.2	DH6-19	JH5	ARRASGWYRQSRYTGWFDPW	VK3-15	98.2	JK4	QQYNNWPGLSF
		RUPA-156	VH3-48	91.3	DH1-26	JH4	ARDGRKDSGSFLDHW	VK1-5	93.9	JK1	QQFDTYWTF
		RUPA-164	VH4-59	95.1	DH2-2	JH6	ARDRVVGSYYYGMDVW	VK3-20	95.0	JK1	QHYGGSPRETF
		RUPA-165	VH4-34	95.1	DH6-19	JH5	ARRSSGYYRGSRYTGWLDPW	VK3-15	94.6	JK4	QQYNNWPGLTF
	2	RUPA-25	VH1-8	91.6	DH3-10	JH5	VRGVGSYYGSGGWFDP	VL1-44	93.8	JL3	ATWDASLKRRV
		RUPA-34	VH3-53	92.7	DH3-22	JH4	VRDSRGWGYFDD	VK3-20	95.5	JK1	QQYQNSPWT
		RUPA-35	VH1-8	94.6	DH3-10	JH5	SRGVGTYHGSGGWFDP	VL1-44	95.5	JL3	ATWDDSLKRGV
		RUPA-41	VH1-8	93.9	DH3-10	JH5	ARGVGTYHGAGGWFDP	VL1-44	94.8	JL3	ATWDDSLKRGV
		RUPA-43	VH1-8	93.6	DH3-10	JH5	ARGVGSYHGSGGWLDP	VL1-44	94.5	JL3	GTWDDSLKRRV
		RUPA-49	VH1-8	90.5	DH3-10	JH5	ARGVGTYHGSGGWFDP	VL1-44	95.5	JL3	ATWDDSLKRGV
		RUPA-53	VH1-8	93.6	DH3-10	JH5	ARGVGSYHGSGGWLDP	VK1-33	98.9	JK4	QQYDNLPLT
		RUPA-63	VH1-8	92.2	DH3-10	JH5	ARGVGTYYGSEGWLDP	VL1-44	95.2	JL3	AAWDDSLKRGV
		RUPA-65	VH1-8	93.2	DH3-10	JH5	ARGVGTYHGSGGWFDP	VL1-44	97.9	JL3	ATWDDSLKRGM
		RUPA-68	VH1-8	93.9	DH3-10	JH5	ARGVGTYHGAGGWFDP	VL1-44	95.5	JL3	ATWDDSLKRGV
		RUPA-74	VH1-8	90.2	DH3-10	JH5	ARGVGSLYGSGGWIDP	VL1-44	94.5	JL3	ETWDDSLKRRV
		RUPA-81	VH3-53	88.7	DH3-10	JH4	ARDVRGWGYLDF	VK3-20	95.5	JK1	QQYGYSPWT
		RUPA-83	VH1-18	91.9	DH4-11	JH5	ARDSRSSQLLQKFDL	VK1-39	92.3	JK1	QQSFSSPT
		RUPA-84	VH1-8	93.2	DH3-10	JH5	ARGVGTYYGSEGWLDP	VL1-44	95.5	JL3	ATWDDSLKRGV
		RUPA-86	VH1-2	95.2	DH3-16	JH6	ARLVAYDQGLDV	VK3-15	98.6	JK2	QQYNNWPPLYT
		RUPA-87	VH1-2	93.2	DH6-19	JH4	ARVVAAEGYFDY	VK1-12	97.9	JK4	QQANSFPLT
		RUPA-88	VH1-8	91.2	DH3-10	JH5	ARGVGSYHGSGGWLDP	VL1-44	94.8	JL3	ATWDDSLKRRV
		RUPA-90	VH3-7	94.9	DH2-2	JH6	AREGDVFVAPSAVRKGGVYGMDV	VK2-28	99.3	JK3	MQALQTPYRVT
		RUPA-91	VH1-8	93.2	DH3-10	JH5	ARGVGTYHGSEGWLDP	VL1-44	95.2	JL3	ATWDGSLKKGV
		RUPA-92	VH1-8	95.6	DH3-10	JH5	ARGVGTYRGSGGWFDP	VL2-11	96.2	JL2	CSYAGNYIEI
		RUPA-99	VH1-8	90.5	DH3-10	JH5	ARGVGSYHGSGGWLDP	VL1-44	94.1	JL3	ATWDDSLKRRV
		RUPA-104	VH1-8	95.3	DH4-17	JH4	ARGVTTPDYFDS	VK1D-12	95.1	JK5	LQANSLPVT
		RUPA-105	VH1-8	89.9	DH3-10	JH5	VRGVGSYYGSGGWLDP	VL1-44	93.4	JL3	ATWDASLKRRV
		RUPA-108	VH3-53	90.7	DH3-22	JH4	VRDSRGWGYFDD	VK3-20	94.8	JK1	QQYQNSPWT
		RUPA-111	VH3-48	91.6	DH1-26	JH4	ARDGRKDSGSFLDH	VK1-5	95.8	JK1	QQFDTYWT
		RUPA-113	VH1-8	91.6	DH3-10	JH5	TRGVGTYHGAGGWFDP	VL1-44	96.9	JL3	ATWDDSLERGV
		RUPA-143	VH1-18	66.7	DH3-16	JH6	ARANWYDNVWQEYFYGMDVW	VK1-5	90.3	JK1	QHYDAYPWTF
		RUPA-144	VH1-8	92.7	DH3-10	JH5	ARGVGTYYGSEGWLDPW	VL1-44	95.4	JL3	ATWDDSLKRGVF
		RUPA-147	VH1-8	91.7	DH3-10	JH5	VRGVGSYYGSGGWFDPW	VL1-44	93.7	JL3	ATWDASLKRRVF
		RUPA-148	VH1-8	91.7	DH3-10	JH5	VRGVGSYYGSGGWFDPW	VL1-44	93.0	JL3	ATWDASLKRRVF

		RUPA-149	VH3-30	91.3	DH3-3	JH6	AKDKVFGVVANHHQYGLDVW	VK1-39	88.5	JK2	QQSYTTPHFTF
		RUPA-150	VH4-34	90.5	DH6-19	JH5	ARRSSGYYRGSRYTGWFDPW	VK3-15	97.9	JK1	QQYNNWPPPWTF
		RUPA-151	VH1-8	91.0	DH3-10	JH5	ARGVGTYHGSEGWLDPW	VL1-44	94.0	JL3	CAAWDDSLKRGVF
		RUPA-153	VH4-31	69.4	DH3-10	JH4	ARAVGVGRYFGYFPSW	VK3-15	97.5	JK1	QQYNNWPPPWTF
		RUPA-160	VH1-8	91.3	DH3-10	JH5	VRAVGSYYGSGGWFDPW	VL1-44	94.7	JL3	ATWDDSMKREVF
		RUPA-161	VH1-8	91.3	DH3-10	JH5	ARGVGTYHGSGGWFDPW	VL1-44	96.1	JL3	ATWDDSLKRGVF
	3	RUPA-29	VH3-33	93.9	DH4 or 15-4a	JH4	ARDFAHGAFYPVD	VL3-10	96.8	JL3	YSSDTSANYWV
		RUPA-42	VH4-31	95.0	DH5-12	JH4	ARAGLVAAIRGSRRGQQGDYFDH	VL3-1	94.0	JL2	QAWDSSTGV
		RUPA-44	VH4-39	96.3	DH3-22	JH4	ARHGGSTGMKVVVIAPPDY	VK1-5	95.4	JK2	QQYHSYPYT
		RUPA-47	VH1-18	89.2	DH3-16	JH4	VRGGRDSPILGGY	VK3-20	95.8	JK4	HQYGSSPLT
		RUPA-50	VH3-30	90.2	DH1-26	JH4	AKDRAHSMHYFEY	VL3-10	95.1	JL2	YSRDGSGDQKNI
		RUPA-54	VH3-30	94.6	DH2-2	JH5	AKDSDNDCTATSCYTVS	VL3-10	96.2	JL2	YSTDSRTDHGGI
		RUPA-57	VH3-30	93.9	DH3-22	JH4	AKSNDHYDNSGYFVY	VL3-10	96.2	JL3	YSRDSSNTQRV
		RUPA-61	VH1-46	93.5	DH5-24	JH6	ARVRKRDAYNNRNWGSVFGGMDV	VL2-8	97.0	JL3	SSFGGSNNLV
		RUPA-70	VH4-31	96.6	DH5-12	JH4	ARTGLVAAIRGSRRGQQGDYFDY	VL3-1	97.2	JL2	QAWDSNTGV
		RUPA-72	VH3-74	93.8	DH2-8	JH6	ARAGCSNGVCPPRYYYHGMDV	VK3-20	96.9	JK1	QHYGNSRVT
		RUPA-89	VH3-23	92.2	DH3-9	JH5	AKEGILRPFDP	VL2-14	96.2	JL1	SSYTSITYHHV
		RUPA-100	VH3-30	96.6	DH2-2	JH4	AKGKAHSCVSTSCYTDF	VL3-10	97.9	JL2	YSTDSSYNHRGV
		RUPA-106	VH3-23	95.9	DH2-8	JH4	AKDTHSNILYYLFDY	VK3-20	97.2	JK1	HQYGISPAT
		RUPA-117	VH4-39	96.3	DH3-22	JH4	ARHGGSTGMKVVVIAPPDY	VK1-5	94.7	JK2	QQYHSYPYT
	?	RUPA-48	VH3-9	96.6	DH2-2	JH6	AKDGGYCSGIRCYHLRGMDV	VK3-11	98.3	JK2	QQRSSWPPYT
		RUPA-51	VH1-18	92.8	DH5-12	JH6	ARGGDRGDGPRFYYYGLDV	VK3-20	94.7	JK1	SLYGRSSWT
		RUPA-67	VH4-4	92.5	DH2-2	JH6	ARDVSPEECVSTKCYRRPGYMDV	VK1-39	95.1	JK1	QQSYTTPRT
		RUPA-80*	VH1-8	99.7	DH2-2	JH5	ARGLPKIYIRGIWFDP	VL3-9	99.3	JL2	QVWDTSTRGV
		RUPA-118*	VH4-4	94.5	DH2-2	JH6	ARDLSPEECVSTKCYRRPGYMDV	VK1-39	95.5	JK1	QQSYTTPRT
		RUPA-154	VH1-2	92.4	DH6-25	JH6	ARLLYSRAVYYYGMDVW	VL3-21	96.8	JL1	QVWDSSSDHYVF
		RUPA-158	VH1-18	93.1	DH5-12	JH6	ARGGDRGDGPRYYYYGLDVW	VK3-20	93.6	JK1	SLYGRSSWTF
U	1	RUPA-130	VH3-15	93.7	DH1-14	JH4	TTDSWAGEVGNYYFDN	VL2-23	95.9	JL1	CSYSKSSTYV
	3	RUPA-140	VH3-23	93.6	DH3-3	JH4	AKVGGRSIFGEATDY	VK1-39	94.4	JK4	QQTYTLPLI
	?	RUPA-141	VH3-7	94.9	DH6-19	JH3	ARDNGARYSGVWYDGWYDAFDI	VK1-6	97.2	JK1	LQDYNYPRT

Table S3. Data collection and refinement statistics for the Pfs48/45-D3-RUPA-29 Fab-RUPA-44 Fab co-crystal structure. Related to Figure 4.

	Pfs48/45-D3-RUPA-29 Fab-RUPA-44 Fab
**Wavelength (Å)**	1.033190
**Space group**	I23
**Cell dimensions**	
**a, b, c (Å)**	223.1 223.1 223.1
**α, β, γ (°)**	90 90 90
**Resolution (Å)**	29.81 - 2.86 (2.91 - 2.86)*
**No. molecules in ASU**	1
**No. unique observations**	42,583 (2126)
**Multiplicity**	40.8 (41.0)
**R_{meas}[1] (%)**	18.3 (312.1)
**R_{pim}[2] (%)**	2.5 (14.6)
**<I/sigma I>**	23.2 (1.5)
**$CC_{1/2}$ (%)**	99.9 (61.2)
**Completeness (%)**	99.9 (100)
**Refinement Statistics**	
**Non-hydrogen atoms**	9,216
**Macromolecule**	9,202
**Water**	0
**Hetero atom**	14
**R_{factor}[3]/ R_{free}[4] (%)**	18.5/ 22.3
**Rms deviations from ideality**	
**Bond lengths (Å)**	0.002
**Bond angle (°)**	0.54
**Ramachandran plot**	
**Favoured regions (%)**	96.0
**Allowed regions (%)**	4.0
**B-factors (Å^2)**	
**Wilson B-factor**	85.7
**Average B-factors**	95.2
**Average macromolecule**	95.1
**Average hetero atom**	156.4
**Average water**	-

*Values in parentheses are for the highest-resolution shell.

[1]$R_{meas} = \Sigma_{hkl} \; [N/(N-1)]^{1/2} \Sigma_i \; | I_{hkl,i} - \langle I_{hkl} \rangle | \; / \; \Sigma_{hkl} \; \langle I_{hkl} \rangle$

[2]$R_{pim} = \Sigma_{hkl} \; [1/(N-1)]^{1/2} \Sigma_i \; | I_{hkl,i} - \langle I_{hkl} \rangle | \; / \; \Sigma_{hkl} \; \langle I_{hkl} \rangle$

[3]$R_{work} = (\Sigma||F_o| - |F_c||)/(\Sigma|F_o|)$.

[4]R_{free} is calculated for 5% of the data that was not included in refinement.

Table S4. Intermolecular contacts for the Pfs48/45-D3-RUPA-29 Fab and Pfs48/45-D3-RUPA-47 Fab co-complexes. VDW=van Der Waals, HB=Hydrogen Bond, SB=Salt Bridge. **Related to Figure 4.**

**Pfs48/45-D3-RUPA-29 Fab co-complex**			**Pfs48/45-D3-RUPA-47 Fab co-complex**		
RUPA-29 Residue (BSA Å^2)	Interaction Type	Pfs48/45-D3 Residue	RUPA-47 Residue (BSA Å^2)	Interaction Type	Pfs48/45-D3 Residue
**H-Arg30 (29.6)**			**H-Gln 1 (16.2)**		
Arg	VDW	Gln355, Tyr371	Gln	VDW	Glu362
**H-Ser31 (42.7)**			**H-Val 2 (18.3)**		
Ser	VDW	Gln355, Ile369, Tyr371	Val	VDW	Glu362, Glu360
Ser^{O}	HB	Gln355^{NE2}	**H-Gly 26 (5.5)**		
Ser^{OG}	HB	Tyr371^{OH}	Gly	VDW	Glu362
**H-Tyr32 (30.0)**			**H-Tyr 27 (7.7)**		
Tyr	VDW	Ser367, Ile369	Tyr	VDW	Glu360
Tyr^{OH}	HB	Ser367^{O}	**H-Asn 31 (13.5)**		
**H-Trp52 (16.0)**			Asn	VDW	Gln 358
Trp	VDW	Asp351	Asn^{O}	HB	Gln358^{NE2}
**H-Tyr52A (66.2)**			**H-Phe 32 (53.6)**		
Tyr	VDW	Asp351, Phe354, Gln355, Tyr371	Phe	VDW	Gln358, Ser367, Glu360, Asn368
Tyr^{OH}	HB	Asp351^{OD2}	**H-Tyr 53 (25.7)**		
**H-Asp53 (3.1)**			Tyr	VDW	Ser374
Asp	VDW	Asp351	**H-Arg94 (46.4)**		
**H-Phe96 (54.1)**			Arg	VDW	Ser367, Glu365, Glu360, Asn368
Phe	VDW	Ile349, Tyr357, Glu365	Arg^{NH1}	SB	Glu360^{OE2}
**H-Ala97 (25.0)**			Arg^{NE}	HB	Ser367^{OG}
Ala	VDW	Ile349, Tyr357, Ile369	Arg^{NH2}	HB	Asn368^{OD1}
**H-His98 (67.7)**			Arg^{NH2}	HB	Ser367^{OG}
His	VDW	Ile348, Ile349, Pro350, Asp351, Gln 355, Ile369	Arg^{NH2}	HB	Glu360^{OE1}
**H-Phe100A (47.2)**			**H-Gly95 (4.7)**		
Phe	VDW	Asp347, Ile348, Ile349	Gly	VDW	Ser367
**L-Leu28 (4.8)**			**H-Gly96 (17.6)**		
Leu	VDW	Lys416	Gly	VDW	Ser367, Ile369
**L-Pro29 (18.5)**			**H-Arg97 (89.5)**		
Pro	VDW	Asp321, Lys416	Arg	VDW	Gln358, Val370, Ile369, Ser367, Ser374, Gln375
**L-Lys30 (54.9)**			Arg^{NH1}	HB	Gln375^{OE1}
Lys	VDW	Asp415, Lys416	Arg^{NH2}	HB	Gln375^{OE1}
Lys^{O}	HB	Lys416^{N}	**H-Asp98 (67.8)**		
**L-Lys31 (36.8)**			Asp	VDW	Ile369, Tyr371, Val370, Ser374, Gln375
Lys	VDW	Asp347, Asp415, Lys416	Asp^{OD2}	HB	Tyr371^{N}
**L-His32 (64.0)**			**H-Ser99 (32.6)**		
His	VDW	Lys413, Asp415, Lys416	Ser	VDW	Tyr371, Gln355
**L-Tyr34 (9.0)**			**H-Ile100A (60.3)**		
Tyr	VDW	Lys413	Ile	VDW	Pro366, Ile369, Ser367, Gln 355
Tyr^{OH}	HB	Lys413^{NZ}	**H-Leu100B (11.5)**		
**L-Tyr49 (27.8)**			Leu	VDW	Pro366, Ser367

Tyr	VDW	Leu364, Glu365	**H-Gly100C (23.6)**		
Tyr^{OH}	HB	Glu365^{OE2}	Gly	VDW	Ser367, Glu365, Pro366
**L-Asp50 (5.9)**			**H-Gly101 (10.7)**		
Asp	VDW	Lys413	Gly	VDW	Glu365
Asp^{OD1}	SB	Lys413^{NZ}	Gly^{N}	HB	Glu365^{OE2}
**L-Asp51 (9.7)**			**H-Tyr102 (51.5)**		
Asp	VDW	Lys416	Tyr	VDW	Glu365, Ser367, Glu362, Glu363
Asp^{OD2}	SB	Lys416^{NZ}	Tyr^{N}	HB	Glu365^{OE2}
**L-Lys53 (34.6)**			**K-Arg29 (54.1)**		
Lys	VDW	Leu364, Glu365	Arg	VDW	Asp351, Phe354, Gln355,
**L-Ser66 (5.9)**			Arg^{NH1}	SB	Asp351^{OD2}
Ser	VDW	Lys416	Arg^{NH1}	HB	Gln355^{OE1}
Ser^{OG}	HB	Lys416^{NZ}	Arg^{NH2}	HB	Gln355^{OE1}
**L-Thr93 (30.5)**			**K-Ser30 (37.2)**		
Thr	VDW	Asp347	Ser	VDW	Pro350, Asp351, Ile348
**L-Ser94 (9.1)**			**K-Met31 (36.9)**		
Ser	VDW	Asp390	Met	VDW	Asp347
			**K-Tyr32 (68.5)**		
			Tyr	VDW	Pro350, Asp351, Gln355, Ile369
			Tyr^{OH}	HB	Asp351^{N}
			Tyr^{OH}	HB	Gln355^{OE1}
			**K-Leu46 (25.4)**		
			Leu	VDW	Glu365, Pro366
			**K-His49 (55.4)**		
			His	VDW	Tyr357, Leu364, Glu365, Pro366
			**K-Ser52 (37.4)**		
			Ser	VDW	Lys413, Asp347
			Ser^{OG}	HB	Lys413^{NZ}
			**K-Ile53 (65.6)**		
			Ile	VDW	Ile349, Tyr357, Lys413
			**K-Thr56 (35.9)**		
			Thr	VDW	Glu363
			**K-Tyr91 (31.9)**		
			Tyr	VDW	Ile369, Pro366

Table S5. Binding affinities of RUPA-29, RUPA-47, RUPA-44, and RUPA-117 Fabs to Pfs48/45-D3 single point mutants. Related to Figures 4 and 5.

Fab	Pfs48/45-D3 constructs	K_D (M) – binding affinity	K_{on} (1/Ms) – on rate	K_{off} (1/s) – off rate	R^2 – the coefficient of determination
**RUPA-29**	Stabilized WT	3.7E-10 ± 4E-11	5.1E+5	2.1E-4	0.982
	K416N	1.1E-7 ± 4E-9	4.2E+5	4.5E-2	0.991
**RUPA-47**	Stabilized WT	3.3E-10 ± 1E-11	3.9E+5	1.2E-4	0.999
	K416N	5.3E-10 ± 2E-11	4.5E+5	2.2E-4	0.997
**RUPA-44**	Stabilized WT	3.9E-9 ± 1E-11	2.2E+5	8.5E-4	0.984
	L314I	3.2E-9 ± 8E-11	2.8E+5	8.9E-4	0.986
	D320H	1.6E-8 ± 3E-10	2.0E+5	3.3E-3	0.986
	S322N	6.8E-9 ± 2E-10	2.1E+5	1.4E-3	0.985
	K416N	3.1E-9 ± 1E-10	3.0E+5	9.2E-3	0.976
**RUPA-117**	Stabilized WT	1.8E-9 ± 5E-11	4.6E+5	7.6E-4	0.984
	L314I	1.9E-9 ± 6E-11	4.2E+5	7.2E-4	0.985
	D320H	7.7E-9 ± 9E-11	4.3E+5	3.3E-3	0.988
	S322N	2.8E-9 ± 5E-11	4.4E+5	1.2E-3	0.981
	K416N	2.4E-9 ± 6E-11	4.7E+5	1.1E-3	0.983

Table S6. Intermolecular contacts for the Pfs48/45-D3-RUPA-117 Fab and Pfs48/45-D3-RUPA-44 Fab co-complexes. VDW=van Der Waals, HB=Hydrogen Bond, SB=Salt Bridge. **Related to Figure 5.**

**Pfs48/45-D3-RUPA-117 Fab co-complex**			**Pfs48/45-D3-RUPA-44 Fab co-complex**		
RUPA-117 Residue (BSA Å^2)	Interaction Type	Pfs48/45-D3 Residue	RUPA-44 Residue (BSA Å^2)	Interaction Type	Pfs48/45-D3 Residue
**H-Arg31 (14.2)**			**H-Arg31 (20.4)**		
Arg	VDW	Leu318	Arg	VDW	Leu318
**H-Pro33 (59.1)**			**H-Pro33 (59.4)**		
Pro	VDW	Leu314, Val319, Leu318,	Pro	VDW	Leu314, Leu318, Val319
**H-Tyr34 (46.5)**			**H-Tyr34 (50.1)**		
Tyr	VDW	Leu318, Ala323, Val319, Asp320	Tyr	VDW	Leu318, Val319, Asp320, Ala323
Tyr^{OH}	HB	Asp320^{N}	Tyr^{OH}	HB	Asp320^{N}
**H-Arg94 (19.1)**			**H-Arg94 (17.6)**		
Arg	VDW	Asp320	Arg	VDW	Asp320
Arg^{NH2}	SB	Asp320^{OD2}	Arg^{NH2}	SB	Asp320^{OD2}
**H-Met 100A (10.4)**			**H-Met 100A (9.8)**		
Met	VDW	Asn328, Ser326	Met	VDW	Asn328
Met^{O}	HB	Asn328^{ND2}	Met^{O}	HB	Asn328^{ND2}
**H-Lys100B (81.5)**			**H-Lys100B (89.3)**		
Lys	VDW	Asn328, Ser326, Cys327, Asp312, Val329, His330	Lys	VDW	Asp312, Ser326, Cys327, Asn328, Val329, His330
Lys^{o}	HB	Asn328^{N}	Lys^{o}	HB	Asn328^{N}
Lys^{o}	HB	Asn328^{ND2}	Lys^{o}	HB	Asn328^{ND2}
Lys^{NZ}	HB	Asn328^{O}	Lys^{NZ}	HB	Asn328^{O}
Lys^{NZ}	SB	Asp312^{OD2}	Lys^{NZ}	SB	Asp312^{OD2}
**H-Val100C (89.9)**			**H-Val100C (91.7)**		
Val	VDW	Ser326, Asn328, Cys327, Leu314, Asp312, Ser313	Val	VDW	Asp312, Ser313, Leu314, Ser326, Cys327, Asn328
**H-Val100D (39.7)**			**H-Val100D (40.1)**		
Val	VDW	Ser326, His324, Ile325	Val	VDW	Leu314, His324, Ile325, Ser326
Val^{N}	HB	Ser326^{O}	Val^{N}	HB	Ser326^{O}
Val^{O}	HB	Ser326^{N}	Val^{O}	HB	Ser326^{N}
**H-Val100E (45.0)**			**H-Val100E (48.2)**		
Val	VDW	His324, Leu314, Ile325, Val319, Ala323	Val	VDW	Leu314, Val319, Ala323, His324, Ile325
**H-Ile100F (53.3)**			**H-Ile100F (48.5)**		
Ile	VDW	Ile325, Ala323, His324, Ser322, Ser326	Ile	VDW	Ser322, Ala323, His324, Ile325, Ser326
Ile^{O}	HB	His324^{N}	Ile^{O}	HB	His324^{N}
Ile^{N}	HB	His324^{O}	Ile^{N}	HB	His324^{O}
**H-Ala100G (12.6)**			**H-Ala100G (15.7)**		
Ala	VDW	Ser322, Ala323, His324	Ala	VDW	Ser322, Ala323, His324
**H-Pro100H (24.6)**			**H-Pro100H (21.7)**		
Pro	VDW	Ser322	Pro	VDW	Ser322
**H-Asp101 (19.5)**			**H-Asp101 (21.8)**		

Asp	VDW	Ser322, Asp320	Asp	VDW	Asp320, Ser322
**H-Tyr102 (6.7)**			**H-Tyr102 (9.9)**		
Tyr	VDW	Asp320	Tyr	VDW	Asp320
**K-Ser28 (26.3)**			**K-Ser28 (27.8)**		
Ser	VDW	Ser361	Ser	VDW	Glu362
Ser^{OG}	HB	$Ser361^{O}$			
**K-Ser30 (24.3)**			**K-Ser30 (38.4)**		
Ser	VDW	Glu363	Ser	VDW	Glu362, Glu363, Leu364
Ser^{OG}	HB	$Glu363^{OE2}$	Ser^{OG}	HB	$Glu363^{O}$
**K-Ile31 (30.5)**			**K-Ser31 (34.7)**		
Ile	VDW	Glu363	Ser	VDW	Glu363, Leu364
Ile^{N}	HB	$Glu363^{OE2}$	Ser^{N}	HB	$Glu363^{O}$
**K-Trp32 (67.0)**			**K-Trp32 (54.9)**		
Trp	VDW	His324, Tyr420	Trp	VDW	His324
**K-Leu46 (11.1)**			**K-Leu46 (10.7)**		
Leu	VDW	Ser322	Leu	VDW	Ser322
**K-Tyr49 (76.0)**			**K-Tyr49 (52.7)**		
Tyr	VDW	Ser322, Asp321, Lys416	Tyr	VDW	Asp321, Ser322
**K-Leu54 (6.6)**			**K-Lys50 (61.6)**		
Leu	VDW	Lys416	Lys	VDW	Asp321, Ser322, Ala323, His324, Leu364
**K-Gln55 (7.2)**			**K-Gln55 (7.6)**		
Gln	VDW	Ser322	Gln	VDW	Ser322
**K-Gly68 (10.0)**			**K-Tyr91 (8.5)**		
Gly	VDW	Glu362	Tyr	VDW	Ser322
**K-Tyr91 (13.5)**					
Tyr	VDW	Ser322			

References

[S1] Roeffen, W., Teelen, K., van As, J., vd Vegte-Bolmer, M., Eling, W., and Sauerwein, R. (2001). Plasmodium falciparum: production and characterization of rat monoclonal antibodies specific for the sexual-stage Pfs48/45 antigen. Exp Parasitol 97, 45-49. 10.1006/expr.2000.4586
